# Genotypic antimicrobial resistance assays for use on *E*. *coli* isolates and stool specimens

**DOI:** 10.1371/journal.pone.0216747

**Published:** 2019-05-10

**Authors:** Suporn Pholwat, Jie Liu, Mami Taniuchi, Rattapha Chinli, Tawat Pongpan, Iyarit Thaipisutikul, Parntep Ratanakorn, James A. Platts-Mills, Molly Fleece, Suzanne Stroup, Jean Gratz, Esto Mduma, Buliga Mujaga, Thomas Walongo, Rosemary Nshama, Caroline Kimathi, Suporn Foongladda, Eric R. Houpt

**Affiliations:** 1 Division of Infectious Diseases and International Health, Department of Medicine, University of Virginia, Charlottesville, Virginia, United States of America; 2 Department of Microbiology, Faculty of Medicine Siriraj Hospital, Mahidol University, Bangkok, Thailand; 3 Swine Veterinarian Service, Charoen Pokphand Foods PCL, Bangkok, Thailand; 4 Faculty of Veterinary Science, Mahidol University, Nakhonpathom, Thailand; 5 Kilimanjaro Clinical Research Institute, Moshi, Tanzania; 6 Haydom Lutheran Hospital, Haydom, Tanzania; Nitte University, INDIA

## Abstract

Antimicrobial resistance (AMR) is an emerging public health problem and methods for surveillance are needed. We designed 85 sequence-specific PCR reactions to detect 79 genes or mutations associated with resistance across 10 major antimicrobial classes, with a focus on *E*. *coli*. The 85 qPCR assays demonstrated >99.9% concordance with sequencing. We evaluated the correlation between genotypic resistance markers and phenotypic susceptibility results on 239 *E*. *coli* isolates. Both sensitivity and specificity exceeded 90% for ampicillin, ceftriaxone, cefepime, imipenem, ciprofloxacin, azithromycin, gentamicin, amikacin, trimethoprim/sulfamethoxazole, tetracycline, and chloramphenicol phenotypic susceptibility results. We then evaluated the assays on direct stool specimens and observed a sensitivity of 97% ± 5 but, as expected, a lower specificity of 75% ± 31 versus the genotype of the *E*. *coli* cultured from stool. Finally, the assays were incorporated into a convenient TaqMan Array Card (TAC) format. These assays may be useful for tracking AMR in *E*. *coli* isolates or directly in stool for targeted testing of the fecal antibiotic resistome.

## Introduction

Antimicrobial resistance (AMR) is a critical public health issue. Antimicrobial-resistant infections can require prolonged treatments, extend hospital stays, and result in greater disability and death compared with susceptible infections [1]. An objective of the World Health Organization (WHO) global action plan on AMR is to strengthen the evidence base through surveillance [2]. Phenotypic culture-based antimicrobial susceptibility testing (AST) is routinely used, however it requires culture and lacks resistance gene information, such as mutations in chromosomal genes or the presence of mobile genetic elements which harbor AMR genes [3–5]; such genotypic information offers useful resolution for epidemiologic purposes, such as tracking the spread of CTX-M [6]. Furthermore, assays that can work in direct stool are advantageous because this specimen is readily accessible compared with those of invasive sites.

We designed and developed 85 genotypic assays primarily targeting Enterobacteriaceae since antibiotic resistance in these bacteria is a particularly threat [1, 7, 8]. We focused on *E*. *coli* because this was the most frequently reported bacteria in the WHO global antimicrobial resistance surveillance system (GLASS) [9] and has been associated with the greatest mortality and morbidity [10]. The assays covered 10 important antimicrobial classes used in human and veterinary medicine including penicillins, cephalosporins, carbapenems, fluoroquinolones, macrolides, aminoglycosides, polymyxins, folate pathway inhibitors, tetracyclines, and phenicols. Here we demonstrate the performance of these assays versus sequencing, compare genotypic results to phenotypic AST, and evaluate the utility of the assays on direct stool.

## Materials and methods

### Bacterial isolates

For validation we tested a variety of both retrospectively and prospectively collected bacterial isolates, including 201 isolates from the Food and Drug Administration and Centers for Disease Control and Prevention Antibiotic Resistance Isolate Bank (FDA-CDC AR bank, CDC, Atlanta, GA, USA), 15 isolates from Antibacterial Resistance Leadership Group (ARLG, Durham, NC, USA), and 20 isolates from American Type Culture Collection (ATCC, Manassas, VA, USA), all of which had been previously sequenced. These isolates represented a range of species, mostly from Enterobacteriaceae (S1 Table). The AMR gene accession numbers provided by the resources are summarized in S2 and S3 Tables. Additionally, we used 81 *E*. *coli* isolates from human stool from Tanzania (Haydom Lutheran Hospital, Haydom), collected as part of the Etiology, Risk Factors and Interactions of Enteric Infections and Malnutrition and the Consequences for Child Health and Development (MAL-ED) birth cohort study [11] to yield a distribution of phenotypically resistant isolates. We also used 107 *E*. *coli* isolates from swine feces which were prospectively collected starting February 2018 for an AMR monitoring study in Thailand (Department of Microbiology, Faculty of Medicine Siriraj Hospital, Mahidol University, Bangkok). This allowed us to obtain at least several resistant bacterial isolates for each antimicrobial agent.

### Stool specimens

Two hundred and twenty direct stool specimens were used, including 70 human stool samples from Tanzania (Haydom Lutheran Hospital, Haydom) also collected as a part of the MAL-ED study. The MAL-ED study was reviewed and approved by the National Institute for Medical Research, Tanzania and the University of Virginia Institutional Review Board (IRB), and informed consent was obtained from the parents or legal guardians of all subjects. One hundred and fifty consecutive swine stool samples from Thailand (Department of Microbiology, Faculty of Medicine Siriraj Hospital, Mahidol University, Bangkok) were collected in February 2018. Animal specimen collection protocol no. 013/2561 was reviewed and approved by Siriraj Animal Care and Use Committees, Siriraj Hospital, Mahidol University. For culture, stool samples were streaked on MacConkey agar and incubated at 35 ± 2°C for 18–24 hour. Five to ten suspected *E*. *coli* colonies were screened by using *E*. *coli* specific PCR assay then confirmed *E*. *coli* colonies were pooled and stored in preservative media at -70°C. Prior to AST, bacteria were subcultured on blood agar (TSA w/ 5% sheep blood, Thermo Scientific, NY, USA) at 35 ± 2°C for 18–24 hours.

### DNA extraction

Genomic DNA from direct stool was extracted using the QIAamp Fast DNA Stool mini kit (Qiagen, Valencia, CA, USA) following the manufacturer’s instructions. Bacterial DNA was extracted by resuspending bacterial colonies in 200 μl TE buffer (10mM Tris-HCl, 1mM EDTA, pH 7.5) or from 500 μl of 0.5 McFarland standard bacterial suspension prepared for phenotypic antimicrobial susceptibility test by centrifugation at 5000x g for 10 min, followed by resuspending the bacterial pellet with 200 μl TE buffer. The bacterial suspensions were incubated at 95°C for 15 min followed by centrifugation at 5000x g for 10 min. The supernatant was stored at -20°C to be used as DNA template.

### PCR Assay development

The primers for amplification of 80–150 bp products and TaqMan probes were designed using Primer Express3 (Applied Biosystems, Life Technologies Corporation, Carlsbad, CA, USA) and online available tool Primer3 (http://bioinfo.ut.ee/primer3/) or adopted from published sources (S4 Table). For Sanger sequencing confirmation, primers that amplified longer products (400–800 bp) were designed using primer3 (http://bioinfo.ut.ee/primer3) (S5 Table). The in silico specificity of primers and probes were tested by using Basic Local Alignment Search Tool (BLAST; https://blast.ncbi.nlm.nih.gov/Blast.cgi). Optimization of conditions and specificity testing of AMR-PCR assays was performed using 384 well plates on the ViiA7 platform (Applied Biosystems, Life Technologies Corporation). Each assay was amplified in duplex (see pairings in S4 Table). Primer/probe sets (final concentrations of 0.9 μM and 0.25 μM for primers and probes, respectively) were assayed in a 5 μl PCR mixture containing 2.5 μl of 2x PCR buffer, 0.2 μl of 25x PCR enzyme of AgPath-ID-PCR kit (Applied Biosystems, Life Technologies Corporation), 0.89 μl nuclease free water, and 1 μl of genomic DNA. Cycling conditions included an initial denaturation at 95°C for 10 min, followed by 40 cycles of denaturation at 95°C for 15 sec and annealing/extension at 60°C for 1 min. The positive control sources included either well-characterized bacterial isolates or synthetic fragment/plasmid controls (Genewiz Inc., South Plainfield, NJ, USA). Synthetic positive control plasmids were constructed (Genewiz Inc.) if neither the genomic material nor the relevant bacterium was available; this included CTX-M8, CMY-1, FOX, GES, gyrA87G-*E*.*coli*, gyrA87N-Y-*Salmonella* spp., and mcr-2. A synthetic positive control plasmid was also constructed (Genewiz Inc.) to contain the primer and probe regions of all 85 targets and used as a positive control for evaluating analytical performance. Genomic DNA of *E*. *coli* ATCC 25922 was used as negative control, and nuclease-free water was used as a no-template control.

### PCR assay evaluation

AMR-PCR assay efficiency and linearity were first determined on the 384 well plate format and subsequently on the TaqMan array card format. For the 384 well plate, the synthetic positive control plasmids (Genewiz Inc.) which contained primer/probe regions of all targets were 10-fold serially diluted in a range of 10^7^ to 1 copy/μl then 1 μl of diluted samples was tested in each 5 μl reaction in triplicate. For the array card format, since the volume of DNA used in the array card is 5-fold lower (0.2 μl/reaction), dilutions of positive control plasmids were prepared in a range of 5x10^7^ to 5 copy/μl to ensure equivalence on both formats. Twenty microliter of each diluted sample was tested in triplicate by mixing with PCR reagents to a total 100 μl then loaded into an array card. The limit of detection (LOD) and precision (repeatability and reproducibility) were determined by spiking positive control plasmid into donor stool followed by extraction and then amplification on the array card. Repeatability was tested with eight repeats of two samples respectively spiked with a high (10^6^ copies/200 mg stool) and a low (10^4^ copies/200 mg stool) concentration of positive control plasmid. Reproducibility was tested with 10 identically spiked samples for each concentration (10^6^ and 10^4^ copies/200 mg stool were interrogated) that were extracted and assayed over 5 days. LOD was defined as the lowest concentration at which the target could be detected in all 10 spiked samples. When comparing the performance of the AMR assays against previously-sequenced bacterial isolates, any discrepancies between PCR and sequence underwent confirmatory repeat PCR and sequencing.

### Evaluation of TaqMan array card

The TaqMan array card was performed as previously described [12]. Briefly, primer and TaqMan probe oligonucleotides were synthesized and spotted into the microfluidic card by Applied Biosystems (Life Technologies Corporation). Twenty microliters of input DNA was mixed with 50 μl of 2x PCR buffer, 4 μl of 25x PCR enzyme of AgPath-ID-PCR kit (Applied Biosystems, Life Technologies Corporation), and 26 μl of nuclease free water to yield a 100 μl final volume. This mixture was loaded into each port of the card and the card was centrifuged twice at 1,200 rpm for 1 min and then sealed. The loading ports were excised and the full card was inserted into a ViiA7 instrument (Life Technologies Corporation) and run under the same cycling conditions as described above.

### Sanger sequencing

The resistance-associated genes were amplified using primers described in S5 Table. The PCR reaction assembly and cycling conditions were described previously [13]. In brief each 25 μl PCR mixture contained 12.5 μl HotStarTaq master mix (Qiagen), 0.25 μl of the 50 μM forward and reverse primers (final concentration of 0.5 μM), 7 μl nuclease free water, and 5 μl of genomic DNA. PCR was performed on a CFX96 (Bio-Rad, Hercules, CA, USA) and included an initial denaturation step at 95°C for 15 min, followed by 40 cycles of denaturation at 95°C for 30 sec, annealing at 60°C for 30 sec, and extension at 72°C for 30 sec, with a final extension step at 72°C for 10 min. PCR products were analyzed on 2% agarose-gels and verified PCR products were purified using MinElute 96 UF PCR Purification Kit (Qiagen) following the manufacturer’s protocol. The purified PCR products were measured spectrophotometrically, diluted with nuclease free water and mixed with primers then submitted to GeneWiz for DNA sequencing (Genewiz Inc.).

### Phenotypic antimicrobial susceptibility testing

The repository isolates and the isolates from Thailand underwent susceptibility testing by broth microdilution method while isolates from Tanzania were previously tested by disc diffusion for ampicillin (AMP), ampicillin/sulbactam (SAM), cefazolin (CFZ), ceftazidime (CAZ), ceftriaxone (CRO), aztreonam (ATM), cefepime (FEP), cefoxitin (FOX), ertapenem (ETP), ciprofloxacin (CIP), gentamicin (GM), and trimethoprim/sulfamethoxazole (TMP-SMX). All of the isolates were tested by broth microdilution method for imipenem (IPM), azithromycin (AZM), amikacin (AMK), kanamycin (KAN), tetracycline (TET), chloramphenicol (CHL), and colistin (CL) and disc diffusion method was used for streptomycin (STR) on all isolates. All methodologies were performed according to the Clinical and Laboratory Standards Institute (CLSI) protocol [14, 15]. Antimicrobial agents used for broth microdilution were AMP, CFZ, FOX, CRO, CAZ, ETP, CIP, AZM, GM, AMK, KAN, TMP-SMX, TET, CHL, CL (all from Sigma-Aldrich, St. Louis, MO, USA), ATM, IPM, sulbactam (all from AdooQ Bioscience, Irvine, CA, USA) and FEP (Alfa Aesar, Tewksbury, MA, USA). In brief for broth microdilution, antimicrobial agents were 2-fold serially diluted in cation-adjusted Mueller Hinton broth (CAMHB, BBL Mueller Hinton II Broth, Becton Dickinson, Sparks, MD, USA) and 100 μl of each dilution including no-antibiotic control media were dispensed into 96 well round bottom culture plates. Bacterial suspensions were prepared in normal saline and adjusted to 0.5 McFarland standards following diluting at 1:20 in sterile distilled water to obtain 5 x 10^6^ cfu/ml. Then 10 μl of bacterial inoculum was inoculated into 96 well round bottom plates and incubated at 35 ± 2°C for 16–20 hour. Antimicrobial agents used for disc diffusion were AMP (10 μg), SAM (10/10 μg), CFZ (30 μg), FOX (30 μg), CRO (30 μg), CAZ (30 μg), FEP (30 μg), ATM (30 μg), ETP (10 μg), CIP (5 μg), GM (10 μg), STR (10 μg), and TMP-SMX (1.25/23.75 μg) (all from Becton Dickinson). For disc diffusion, the 0.5 McFarland standard bacterial suspensions were dipped by sterile cotton swab and swabs were streaked over the entire Mueller Hinton agar (MHA, BBL Mueller Hinton II Agar, Becton Dickinson) surface. The disc containing antibiotics were placed onto the surface of inoculated agar plate, and incubated at 35 ± 2°C for 16–18 hour. The *E*. *coli* ATCC 25922, and *P*. *aeruginosa* ATCC 27853 (for carbapenem) were used as quality control and the minimal inhibitory concentration (MIC) and zone diameter interpretative standard of CLSI-M100 Ed29 [16] were used for interpretation. The results of standard phenotypic AST and genotypic PCR testing were unblinded to the reader. If there were any discrepancies between PCR and AST then both methods were repeated and the repeat results were considered final (540/568 or 95.1% were identical to the original result). The phenotypic AST results of all isolates are shown in S6 Table.

### Statistical analysis

The sensitivity, specificity, and accuracy of genotypic test methods were analyzed against phenotypic methods as the gold standard. The kappa coefficient (κ) was calculated with GraphPad QuickCalcs (https://www.graphpad.com/quickcalcs/kappa1.cfm) to measure agreement between methods. Receiver-operating characteristic (ROC) analysis was performed with SPSS Statistics Software to define a Ct (quantification cycle) cut-off that optimized sensitivity and specificity.

## Results

### Antimicrobial resistance associated gene targets

We sought to develop assays to detect resistance to the antimicrobial classes commonly used in both human and veterinary medicine, namely penicillins, cephalosporins, carbapenems, fluoroquinolones, macrolides, aminoglycosides, folate pathway inhibitors, tetracyclines, phenicols, and polymyxins. The gene targets were chosen based on previously reported genes or mutations and we prioritized candidates based on global prevalence (S7 Table). Because there are many subgroups of genes (e.g., CTX-M), most assays were designed in conserved regions as group-specific assays (S8 Table). In addition to AMR targets, since a goal was to later evaluate these assays directly on stool specimens we also included *E*. *coli*/*Shigella* spp., *Salmonella* spp., and *Campylobacter* spp. specific assays for fluoroquinolone (in *gyrA* and *parC*) and macrolide resistance (in 23S rRNA), as well as previously published detection assays for these genera [17–20]. Additionally, internal and external controls were included (bacterial 16S rRNA and phocine herpesvirus, respectively). This amounted to PCR assays that included 69 primer pairs and 85 specific probes (S4 Table).

### PCR assay performance versus sequencing

We organized the assays into 42 duplex reactions and 1 singleplex reaction on a 384 well plate using dilutions of positive control plasmid. The overall linearity of the 85 assays was 0.999 ± 0.002 and PCR efficiencies were 96.2% ± 3.9 (S9 Table). The specificity of the assays was tested against 15 other commonly found enteropathogens including *Aeromonas hydrophila*, *Adenovirus*, *Bacteroides fragilis*, *Blastocystis hominis*, *Clostridium difficile*, *Cryptosporidium hominis*, *Entamoeba histolytica*, *Encephalitozoon intestinalis*, *Giardia lamblia*, *Helicobacter pylori*, *Schistosoma mansoni*, *Vibrio cholerae*, *Vibrio parahaemolyticus*, *Yersinia enterocolitica*, and *Yersinia pseudotuberculosis* and no false positives were observed. Assay performance was then tested against 236 previously-sequenced bacterial isolates consisting of several genera and species (S1 Table). The genotypic PCR assays showed 100% sensitivity and >99.9% overall concordance against sequencing, with 11/20060 discrepancies (Table 1).

**Table 1:** Comparison of AMR-PCR assays versus sequencing on bacterial isolates (N = 236).

Targets	No of positive^a^ tested	No of negative^a^ tested	PCR assay result		Concordance (%)	Targets	No of positive^a^ tested	No of negative^a^ tested	PCR assay result		Concordance (%)
			Positive	Negative					Positive	Negative	
Beta lactam genes											
TEM 104E	105	131	105	131	100	TEM 104K	3	233	3	233	100
TEM 164R	106	130	106	130	100	TEM 164SC	2	234	2	234	100
DHA	3	233	3	233	100	TEM 238S	5	231	5	231	100
SHV	68	168	68	167/168	99	SHV 238-240SE-SK	28	208	28	208	100
CTX-M1	43	193	43	193	100	CTX-M8-M25	0	236	0	236	100
CTX-M2-M74	5	231	5	231	100	CTX-M9	6	230	6	229/230	99
PER	3	233	3	233	100	VEB	1	235	1	235	100
CMY1-MOX	0	236	0	236	100	FOX	0	236	0	236	100
CMY2-LAT	37	199	37	199	100	ACT-MIR	14	222	14	222	100
KPC	37	199	37	199	100	GES	0	236	0	236	100
NDM	37	199	37	199	100	VIM	10	226	10	226	100
IMP	5	231	5	231	100	OXA-48	12	224	12	224	100
OXA-1	37	199	37	199	100	OXA-9	30	206	30	205/206	99
Fluoroquinolone genes											
QnrA	3	233	3	233	100	QnrS	8	228	8	228	100
QnrB1	17	219	17	218/219	99	QnrB4	20	216	20	216	100
aac(6’)-lb-104W	68	168	68	168	100	aac(6’)-lb-104R	38	198	38	198	100
gyrA87G-ESh^b^	0	236	0	236	100	aac(6’)-lb-181Y	38	198	38	198	100
QepA	1	235	1	235	100	gyrA87G-Sal^c^	2	234	2	234	100
gyrA83S-Sal^c^	8	228	8	226/228	99	gyrA83FY-Sal^c^	3	233	3	233	100
gyrA87D-Sal^c^	9	227	9	227	100	gyrA87NY-Sal^c^	0	236	0	236	100
gyrA83S-ESh^b^	22	214	22	21	100	gyrA83L-ESh^b^	40	196	40	195/196	99
gyrA87D-ESh^b^	23	213	23	213	100	gyrA87NY-ESh^b^	39	197	39	197	100
parC80S-Sal^c^	9	227	9	226/227	99	parC80I-Sal^c^	2	234	2	234	100
parC80S-ESh^b^	25	211	25	211	100	parC80I-ESh^b^	37	199	37	199	100
gyrA86T-Cj^d^	3	233	3	233	100	gyrA86I-Cj^d^	2	234	2	234	100
gyrA86T-Cc^e^	3	233	3	233	100	gyrA86I-Cc^e^	2	234	2	234	100
Macrolide genes											
23S-2075A-Cp^f^	5	231	5	231	100	23S-2075G-Cp^f^	5	231	5	231	100
ErmB	6	230	6	230	100	mphA	50	186	50	186	100
Aminoglycoside genes											
armA	17	219	17	219	100	rmtB	3	233	3	233	100
aacC1	4	232	4	232	100	aacC2	52	184	52	184	100
aacC4	7	229	7	229	100	aadB	21	215	21	215	100
aphA1	38	198	38	197/198	99	aadA1-2-17	93	143	93	143	100
Folate pathway inhibitor genes											
dfrA1	32	204	32	204	100	dfrA12	40	196	40	196	100
dfrA5-14	38	198	38	198	100	dfrA17	22	214	22	212/214	99
sul1	125	111	125	111	100	sul2	80	156	80	156	100
sul3	8	228	8	228	100						
Tetracycline genes											
tetA	58	178	58	178	100	tetB	25	211	25	211	100
Phenicol genes											
catA1	39	197	39	197	100	catB3	8	228	8	228	100
cmlA	27	209	27	209	100	floR	18	218	18	218	100
Polymyxin genes											
mcr-1	6	230	6	230	100	mcr-2	0	236	0	236	100
Bacterial genera and controls											
*E*.*coli-Shigella*	61	175	61	175	100	*Shigella* spp.	7	229	7	229	100
*Salmonella* spp.	11	225	11	225	100	*C*. *jejuni-coli*	10	226	10	226	100
PhHV	0	236	0	236	100	Bacterial 16S	236	0	236	0	100
Total	2171	17889	2171	17878/17889	99.9						

^a^ Whole genome sequencing or Sanger sequencing

^b^ ESh; *E*.*coli-Shigella* spp.,

^c^ Sal; *Salmonella* spp.,

^d^ Cj; *C*. *jejuni*,

^e^ Cc; *C*. *coli*,

^f^ Cp; *Campylobacter* spp.

Note: isolates that had both mutation and wild-type *gyrA* and/or *parC* were excluded from analysis of fluoroquinolone resistance.

### Correlation between genotypic and phenotypic antimicrobial susceptibility testing

We then evaluated the correlation between genotypic and phenotypic AST on 239 *E*.*coli* isolates. This included a range of susceptible and resistant isolates from FDA-CDC-AR bank (n = 42), ARLG (n = 4), ATCC (n = 5), clinical human isolates (n = 81) and swine isolates (n = 107). This evaluation is based on the necessary but oversimplified assumption that if a resistance-associated gene or mutation was present, at any quantity (Ct cutoff 30), then that isolate would be resistant to that antimicrobial agent, while if such a gene or mutation was absent then the isolate would be susceptible. This comparison showed that the sensitivity for detecting phenotypic resistance ranged between 86% - 100% for 15 antimicrobial agents (i.e., the very major error rates were 0–14%), whereas sensitivity for resistance to cefoxitin, kanamycin, streptomycin, colistin, and ampicillin/sulbactam was lower at 76%, 75%, 72%, 67%, and 43% respectively (Table 2). The specificity of the assays for detecting phenotypic susceptibility ranged between 88% - 100% for all antimicrobial agents (i.e., major error rates 0–12%) except streptomycin and cefazolin (78% and 70%, respectively). Overall, sensitivity and specificity exceeded 90% for ampicillin, ceftriaxone, cefepime, imipenem, ciprofloxacin, azithromycin, gentamicin, amikacin, trimethoprim/sulfamethoxazole, tetracycline, and chloramphenicol phenotypic susceptibility results, with substantial or better kappa (κ) agreement between the two methods (κ = 0.79–0.97). Categorical agreement of the genotypic versus phenotypic method, ignoring intermediate results which cannot be categorized, was greater than 90% for all antimicrobial agents except for ampicillin/sulbactam (66%) and streptomycin (74%).

**Table 2:** Correlation between genotypic (AMR-PCR assay) and phenotypic AST of *E*. *coli* isolates (N = 239).

Antibiotic	Resistant genes	PCR assay	Phenotypic AST^a^			Sens. (%)	Spec. (%)	Categorical agreement (%)	Kappa^b^ (κ)
			R	I	S				
Ampicillin	Class A β-lactamase; TEM, SHV, CTX-M1, CTX-M8, CTX-M9, KPC	Positive	202	1	1	99	97	99	0.97
	Class B β-lactamase; NDM	Negative	1	1	33				
	Class C β-lactamase; CMY2-LAT, ACT-MIR, DHA								
	Class D β-lactamase; OXA-1, OXA-9, OXA-48								
Ampicillin/	Class B β-lactamase; NDM	Positive	45	8	0	43	100	66	0.38
sulbactam	Class C β-lactamase; CMY2-LAT, ACT-MIR, DHA	Negative	59	55	72				
	Class D β-lactamase; OXA-1, OXA-9, OXA-48								
Cefazolin	Class A β-lactamase; TEM, SHV, CTX-M1, CTX-M8, CTX-M9, KPC	Positive	137	53	14	99	70	91	0.75
	Class B β-lactamase; NDM	Negative	2	0	33				
	Class C β-lactamase; CMY2-LAT, ACT-MIR, DHA								
	Class D β-lactamase; OXA-1, OXA-9, OXA-48								
Cefoxitin	Class B β-lactamase; NDM	Positive	31	2	0	76	100	96	0.84
	Class C β-lactamase; CMY2-LAT, ACT-MIR, DHA	Negative	10	13	183				
Ceftazidime	Class A β-lactamase; TEM-ESBL, SHV-ESBL, CTX-M1, CTX-M8, CTX-M9, KPC	Positive	71	6	19	100	88	92	0.82
	Class B β-lactamase; NDM	Negative	0	0	143				
	Class C β-lactamase; CMY2-LAT, ACT-MIR, DHA								
Ceftriaxone	Class A β-lactamase; TEM-ESBL, SHV-ESBL, CTX-M1, CTX-M8, CTX-M9, KPC	Positive	87	0	7	99	95	97	0.93
	Class B β-lactamase; NDM	Negative	1	0	144				
	Class C β-lactamase; CMY2-LAT, ACT-MIR, DHA								
Cefepime	Class A β-lactamase; CTX-M1, CTX-M8, CTX-M9, KPC	Positive	58	9	8	95	95	95	0.88
	Class B β-lactamase; NDM	Negative	3	1	160				
Aztreonam	Class A β-lactamase; TEM-ESBL, SHV-ESBL, CTX-M1, CTX-M8, CTX-M9, KPC	Positive	69	5	20	100	88	91	0.81
	Class C β-lactamase; CMY2-LAT, ACT-MIR, DHA	Negative	0	1	144				
Ertapenem	Class A β-lactamase; KPC	Positive	18	0	0	86	100	99	0.92
	Class B β-lactamase; NDM	Negative	3	2	216				
	Class D β-lactamase; OXA-48								
Imipenem	Class A β-lactamase; KPC	Positive	18	0	0	90	100	99	0.94
	Class B β-lactamase; NDM	Negative	2	0	219				
	Class D β-lactamase; OXA-48								
Ciprofloxacin^c^	gyrA, parC	Mutant	61	0	7	97	94	95	0.89
		Mt + Wt	17	6	12				
		Wild-type	2	17	117				
Azithromycin^d^	ermB, mphA	Positive	78	0	3	95	98	97	0.93
		Negative	4	0	154				
Gentamicin	aacC2, aacC4, aac(6’)-lb, aadB, rmtB	Positive	79	1	4	96	97	97	0.93
		Negative	3	0	152				
Amikacin	aac(6’)-lb, rmtB	Positive	12	0	6	100	97	97	0.79
		Negative	0	0	221				
Kanamycin	aphA1	Positive	51	1	0	75	100	93	0.81
		Negative	17	2	168				
Streptomycin	aadA1-2-17	Positive	107	9	18	72	78	74	0.47
		Negative	41	1	63				
Trimethoprim/	dfrA1, dfrA5-14, dfrA12, dfrA17, sul1, sul2, sul3	Positive	168	0	2	92	96	93	0.83
sulfamethoxazole		Negative	14	0	55				
Tetracycline	tetA, tetB	Positive	172	0	4	99	94	97	0.94
		Negative	2	0	61				
Chloramphenicol	catA1, catB3, cmlA, floR	Positive	112	6	9	99	92	96	0.91
		Negative	1	9	102				
Colistin^e^	mcr-1	Positive	29	0	1	67	99	94	0.76
		Negative	14	0	195				

^a^ Excluded intermediate (I) from analysis

^b^ Strength of the kappa (κ) coefficients: 0.01–0.20 slight; 0.21–0.40 fair; 0.41–0.60 moderate; 0.61–0.80 substantial; 0.81–1.0 almost perfect agreement

^c^ Excluded 35 mixed mutant and wild-type from analysis

^d^ Used interpretative criteria of CLSI M100 29Ed [16] for *Salmonella enterica* Typhi where MIC ≤16 is susceptible and ≥32 is resistant

^e^ Used interpretative criteria of CLSI M100 29Ed [16] for *Pseudomonas aeruginosa* where MIC ≤2 is susceptible and ≥4 is resistant

Sens.; sensitivity, Spec.; specificity

### AMR detection in direct stool specimens

We then sought to evaluate the sensitivity of these AMR-PCR assays on direct stool specimens versus the genotypic pattern of the *E*. *coli* cultured from the stool. The focus on *E*. *coli* was based on its importance as an indicator organism, a member of the stool microbiome, and a reservoir of AMR genes. Comparing results from 220 stool DNA (70 human, 150 swine) versus the paired *E*.*coli* isolates cultured from those stools, using a Ct cut-off of 32 (S1 Fig for ROC analysis), direct genotypic testing of stool predicted the cultured *E*. *coli* genotype with an overall sensitivity of 97% ± 5 across all genes, an overall specificity of 75% ± 31, and an overall accuracy 85% ± 17 (Table 3).

**Table 3:** Comparison of AMR-PCR assay detection in direct stool and paired *E*.*coli* isolates (N = 220).

Targets	N positive by culture^a^	N negative by culture^a^	Direct stool result		Sens. (%)	Spec. (%)	Targets	N positive by culture^a^	N negative by culture^a^	Direct stool result		Sens. (%)	Spec. (%)
			Positive	Negative						Positive	Negative		
Beta lactam genes													
TEM 104E	190	30	190	0/30	100	0	TEM 104K	0	220	0	220	NA	100
TEM 164R	190	30	190	0/30	100	0	TEM 164SC	0	220	0	220	NA	100
DHA	2	218	2	183/218	100	84	TEM 238S	0	220	0	220	NA	100
SHV	2	218	2	181/218	100	83	SHV 238-240SE-SK	0	220	0	205/220	NA	93
CTX-M1	42	178	40/42	143/178	95	80	CTX-M8-M25	1	219	1	211/219	100	96
CTX-M2-M74	0	220	0	218/220	NA	99	CTX-M9	29	191	27/29	139/191	93	73
PER	0	220	0	218/220	NA	99	VEB	2	218	2	151/218	100	69
CMY1-MOX	0	220	0	217/220	NA	99	FOX	0	220	0	220/220	NA	100
CMY2-LAT	8	212	7/8	170/212	87	80	ACT-MIR	4	216	4	178/216	100	82
KPC	0	220	0	220/220	NA	100	GES	0	220	0	196/220	NA	89
NDM	0	220	0	220/220	NA	100	VIM	0	220	0	220	NA	100
IMP	0	220	0	216/220	NA	98	OXA-48	0	220	0	220	NA	100
OXA-1	14	206	11/14	138/206	79	67	OXA-9	0	220	0	220	NA	100
Fluoroquinolone genes													
QnrA	0	220	0	218/220	NA	99	QnrS	126	94	126	54/94	100	57
QnrB1	0	220	0	175/220	NA	79	QnrB4	1	219	1	211/219	100	96
aac(6’)-lb-104W	3	217	3	141/217	100	65	aac(6’)-lb-104R	4	216	4	197/216	100	91
gyrA87G-ESh^b^	2	218	2	217/218	100	99	aac(6’)-lb-181Y	4	216	4	192/216	100	89
gyrA83S-ESh^b^	196	24	195/196	20/24	99	83	gyrA83L-ESh^b^	86	134	77/86	124/134	89	92
gyrA87D-ESh^b^	206	14	206	12/14	100	86	gyrA87NY-ESh^b^	37	183	33/37	180/183	89	98
parC80S-ESh^b^	199	21	197/199	19/21	99	90	parC80I-ESh^b^	30	190	28/30	188/190	93	99
QepA	1	219	1	214/219	100	98							
Macrolide genes													
ermB	39	181	39	57/181	100	31	mphA	70	150	67/70	92/150	96	61
Aminoglycoside genes													
armA	0	220	0	220	NA	100	rmtB	5	215	5	205/215	100	95
aacC1	0	220	0	204/220	NA	93	aacC2	95	125	93/95	81/125	98	65
aacC4	7	213	7	72/213	100	34	aadB	5	215	5	87/215	100	40
aphA1	80	140	80	43/140	100	31	aadA1-2-17	172	48	171/172	7/48	99	15
Folate pathway inhibitor genes													
dfrA1	43	177	43	23/177	100	13	dfrA12	137	83	136/137	52/83	99	63
dfrA5-14	82	138	78/82	80/138	95	58	dfrA17	46	174	36/46	138/174	78	79
sul1	73	147	73	14/147	100	9	sul2	154	66	154	0/66	100	0
sul3	145	75	142/145	65/75	98	87						100	NA
Tetracycline genes													
tetA	177	43	176/177	1/43	99	2	tetB	108	112	108	15/112	100	13
Phenicol genes													
catA1	44	176	41/44	148/176	93	84	catB3	3	217	3/3	173/217	100	80
cmlA	143	77	141/143	68/77	99	88	floR	72	148	71/72	123/148	99	83
Polymyxin genes													
mcr-1	26	194	26	176/194	100	91	mcr-2	0	220	0	192/220	NA	87
Bacterial genera and control													
*E*.*coli-Shigella*	220	0	220	0	100	NA	*Shigella* spp.	0	220	0	220/220	NA	100
Bacterial 16S	220	0	220	0	100	NA							

^a^ *E*. *coli* isolated from paired stool samples

^b^ ESh; *E*.*coli-Shigella* spp.

NA; not applicable, Sens.; sensitivity, Spec.; specificity

### Performance of AMR-TAC

**Fig 1:**
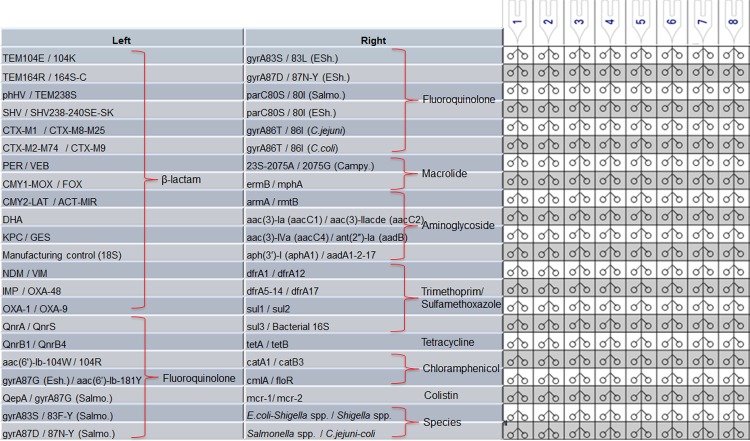
Antimicrobial resistance TaqMan array card (AMR-TAC) layout. The TaqMan array card includes 8 sample ports. Each well was configured and grouped according to antimicrobial resistance associated with those gene targets. Two wells were used for bacterial species/genera detection. The symbol “/” indicates a duplex assay. Because we amplified 42 duplex, 1 singleplex (DHA), and 1 singleplex manufacturing control target, only 44 wells were used out of the 48 well TAC card.

The AMR-PCR assays were then compartmentalized into a TaqMan array card (TAC) format and the analytical PCR performance of each assay was determined (Fig 1). The overall linearity of the 85 targets was 0.999 ± 0.001 and PCR efficiencies were 95.1% ± 2.5 (S10 Table). The limit of detection, defined as lowest copy number that was detected in all 10 extractions/amplification, was 10^4^ copies per 200 mg stool (10 copies per PCR reaction). The coefficient of variant (CV) of Ct values was 3.6% ± 2.0 and 4.7% ± 2.1 for repeatability and reproducibility, respectively. The performance of AMR-TAC was then determined against 122 DNA samples including direct stools (n = 56) and cultured isolates (n = 66). TAC yielded nearly perfect concordance with the plate results: 100% concordance on cultured isolates and 99.6% ± 1.5 sensitivity and 99.2% ± 3.5 specificity on direct stools (Table 4).

**Table 4:** Performance of TaqMan array card (TAC) compared with 384 well PCR plate for AMR detection in direct stool and cultured isolates (N = 122).

Targets	N positive^a^ tested	N negative^a^ tested	TAC result		Sens. (%)	Spec. (%)	Targets	N positive^a^ tested	N negative^a^ tested	TAC result		Sens. (%)	Spec. (%)
			Positive	Negative						Positive	Negative		
Beta lactam genes													
TEM 104E	97	25	97	25	100	100	TEM 104K	3	119	3	119	100	100
TEM 164R	98	24	98	24	100	100	TEM 164SC	3	119	3	119	100	100
DHA	18	104	17/18	103/104	94	99	TEM 238S	3	119	3	119	100	100
SHV	44	78	44	78	100	100	SHV238-240SE-SK	21	101	20/21	101	95	100
CTX-M1	51	71	50/51	70/71	98	99	CTX-M8-M25	15	107	15	106/107	100	99
CTX-M2-M74	6	116	6	116	100	100	CTX-M9	39	83	39	83	100	100
PER	5	117	5	117	100	100	VEB	28	94	28	94	100	100
CMY1-MOX	3	119	3	119	100	100	FOX	0	122	0	122	NA	100
CMY2-LAT	39	83	39	83	100	100	ACT-MIR	26	96	26	96	100	100
KPC	5	117	5	117	100	100	GES	19	103	19	103	100	100
NDM	11	111	11	111	100	100	VIM	3	119	3	119	100	100
IMP	9	113	9	112/113	100	99	OXA-48	3	119	3	119	100	100
OXA-1	42	80	42	80	100	100	OXA-9	10	112	10	112	100	100
Fluoroquinolone genes													
QnrA	5	117	5	117	100	100	QnrS	52	70	52	70	100	100
QnrB1	32	90	32	90	100	100	QnrB4	19	103	19	103	100	100
aac(6’)-lb-104W	52	70	52	69/70	100	99	aac(6’)-lb-104R	30	92	30	92	100	100
gyrA87G-ESh^b^	1	121	1	121	100	100	aac(6’)-lb-181Y	28	94	28	94	100	100
QepA	8	114	8	114	100	100	gyrA87G-Sal^c^	2	120	2	120	100	100
gyrA83S-Sal^c^	4	118	4	118	100	100	gyrA83FY-Sal^c^	3	119	3	119	100	100
gyrA87D-Sal^c^	4	118	4	118	100	100	gyrA87NY-Sal^c^	0	122	0	122	NA	100
gyrA83S-ESh^b^	58	64	58	64	100	100	gyrA83L-ESh^b^	57	65	57	65	100	100
gyrA87D-ESh^b^	61	61	61	61	100	100	gyrA87NY-ESh^b^	33	89	33	89	100	100
parC80S-Sal^c^	4	118	4	118	100	100	parC80I-Sal^c^	2	120	2	120	100	100
parC80S-ESh^b^	61	61	61	61	100	100	parC80I-ESh^b^	27	95	27	95	100	100
gyrA86T-Cj^d^	5	117	5	117	100	100	gyrA86I-Cj^d^	2	120	2	120	100	100
gyrA86T-Cc^e^	2	120	2	120	100	100	gyrA86I-Cc^e^	15	107	15	107	100	100
Macrolide genes													
23S-2075A-Cp^f^	22	100	21/22	100	95	100	23S-2075G-Cp^f^	33	89	33	89	100	100
ermB	50	72	50	72	100	100	mphA	65	57	65	57	100	100
Aminoglycoside genes													
armA	6	116	6	116	100	100	rmtB	13	109	13	109	100	100
aacC1	15	107	15	107	100	100	aacC2	68	54	68	54	100	100
aacC4	41	81	41	80/81	100	99	aadB	44	78	44	78	100	100
aphA1	62	60	62	60	100	100	aadA1-2-17	84	38	83/84	38	99	100
Folate pathway inhibitor genes													
dfrA1	65	57	65	57	100	100	dfrA12	61	61	61	61	100	100
dfrA5-14	60	62	60	60/62	100	97	dfrA17	41	81	41	77/81	100	95
sul1	95	27	95	27	100	100	sul2	83	39	83	39	100	100
sul3	50	72	50	72	100	100							
Tetracycline genes													
tetA	76	46	76	46	100	100	tetB	70	52	70	52	100	100
Phenicol genes													
catA1	45	77	45	77	100	100	catB3	28	94	28	93/94	100	99
cmlA	58	64	58	64	100	100	floR	52	70	52	70	100	100
Polymyxin genes													
mcr-1	30	92	30	92	100	100	mcr-2	9	113	9	113	100	100
Bacterial genera and controls													
*E*.*coli-Shigella*	81	41	81	41	100	100	*Shigella* spp.	5	117	5	117	100	100
*Salmonella* spp.	7	115	7	115	100	100	*C*. *jejuni-coli*	24	98	24	98	100	100
PhHV	56	66	54/56	66	96	100	Bacterial 16S	122	0	122	0	100	NA

^a^ Results on 384 well PCR plate

^b^ ESh; *E*.*coli-Shigella* spp.,

^c^ Sal; *Salmonella* spp.,

^d^ Cj; *C*. *jejuni*,

^e ^Cc; *C*. *coli*,

^f^ Cp; *Campylobacter* spp.

NA; not applicable, Sens.; sensitivity, Spec.; specificity

## Discussion

In this work we developed an extensive menu of qPCR assays to detect AMR-associated genes or mutations for 10 antimicrobial classes that can be used for epidemiologic purposes. The accuracy was almost perfect compared to direct sequencing, with only 0.05% discrepancy. When used on direct stool samples, the PCR assays were sensitive at detecting the AMR genes carried by resident *E*. *coli*. As expected the specificity was lower, presumably because AMR genes in stool derive from any member of bacteria besides *E*. *coli*. Such a high sensitivity assay could be useful as a screening test of the resistome in surveillance specimens such as human and livestock stool or environmental materials for epidemiologic purposes [21, 22]. Of course, further evaluation in this area is needed, as mechanisms of resistance may differ geographically, and we only assessed stool specimens from two countries.

The genotypic-phenotypic correlation on bacterial isolates was good, yielding >90% sensitivity and specificity versus the phenotypic results for ampicillin, ceftriaxone, cefepime, imipenem, ciprofloxacin, azithromycin, gentamicin, amikacin, trimethoprim/sulfamethoxazole, tetracycline, and chloramphenicol across a range of genera. Antimicrobial agents whose genotypic-phenotypic correlation was suboptimal included ampicllin/sulbactam, potentially because we included a limited number of class D β-lactamase (OXA-type) targets or because of other mechanisms of resistance, such as penicillinase hyperproduction, overproduction of constitutive AmpC cephalosporinase, and inhibitor-resistant TEM (IRT) β-lactamase [23]. Cefoxitin resistance was also difficult to detect genotypically (76% sensitivity), perhaps because we only included plasmid mediated AmpC β-lactamase, not chromosomal AmpC, or because we did not test for outer membrane porins [24, 25]. Similarly, for colistin (67% sensitivity) we only included the plasmid-mediated *mcr* gene, while resistance may be due to several other mechanisms [17]. Detecting kanamycin and streptomycin resistance was also of lower sensitivity, perhaps because other targets such as *aph(3’)-IIa*, *strA*, and *strB* [26] were not included. As for the specificity to detect susceptibility, cefazolin was the lowest (70%) and mostly due to *bla*_TEM_ positive but phenotypically susceptible isolates, likely due to low expression. Results for streptomycin and *aadA* were similar. Therefore, if a phenotypic susceptibility result for these drugs is desired, further assay optimization is needed. These drugs aside, however, the assays worked are usable for surveillance purposes for the 11 drugs with >90% sensitivity and specificity: ampicillin, ceftriaxone, cefepime, imipenem, ciprofloxacin, azithromycin, gentamicin, amikacin, trimethoprim/sulfamethoxazole, tetracycline, and chloramphenicol. Certainly, use for clinical care would require commercial development, and major error and very major error rates should be below 3% and 1.5% per CLSI.

If desired, the PCR assays can be used on the TaqMan Array Card format. We have found this to be an easy to perform, rapid, and high-throughput tool. Cost of the TAC reagents (~$50 per specimen) and platform remain a substantial limitation. However the alternatives are costly as well. Sanger sequencing is costly, as are whole genome sequencing technologies, which also requires extensive bioinformatic interpretation [27–29].

In sum, we present a menu of AMR qPCR assays that can be used for tracking AMR in bacterial isolates, primarily Enterobacteriaceae and *E*. *coli*, and also in direct stool specimens for epidemiologic purposes.

## Supporting information

S1 Table Previously-sequenced bacterial isolates. (DOCX)

S2 Table Summary of AMR genes of 236 bacterial isolates. (XLSX)

S3 Table Glossary of AMR genes. (XLSX)

S4 Table Primer and probe sequences of the 42 duplex and 1 singleplex PCR reactions. (DOCX)

S5 Table Sequencing primers. (DOCX)

S6 Table Phenotypic AST results of 239 *E*.*coli* isolates. (XLSX)

S7 Table Antimicrobial agent classes and gene targets included in AMR-TAC. (DOCX)

S8 Table Subgroups or members of group assays. (DOCX)

S9 Table Analytical PCR performance of each assay on 384 well plate format. (DOCX)

S10 Table Analytical performance of antimicrobial resistance TaqMan array card (AMR-TAC). (DOCX)

S1 Fig Scatter plot of difference Ct values. Scatter plot of difference Ct values of 220 direct stools against paired *E*. *coli* isolates results of each target gene associated resistance to β-lactam (A), fluoroquinolone (B and C), Macrolide (D), aminoglycoside (E), trimethoprim/sulfamethoxazole (F), tetracycline (G), chloramphenicol (H), and colistin (I). Receiver Operating Curves (ROC) identified cut-off for optimized positive/negative categorization of direct stool against *E*. *coli* culture isolates for *E*. *coli* specific gene *gyrA* and *parC*, then the same cut-off was applied to all other gene targets which non-*E*. *coli* specific.
(TIF)

S1 ARRIVE guidelines checklist (PDF)
