# Supplementary material for: Genotypic antimicrobial resistance assays for use on E. coli isolates and stool specimens
Source: PLoS One. 2019 May 10;14(5):e0216747. doi: 10.1371/journal.pone.0216747 (PMC6510447; doi:10.1371/journal.pone.0216747)
Supplement: S1 Table — (DOCX) [file pone.0216747.s001.docx]

**S1 Table. Previously-sequenced bacterial isolates.**

| **FDA-CDC AR Bank (Whole genome sequencing)** | | **ARLG (Target (Sanger) sequencing)** | |
| --- | --- | --- | --- |
| **Bacterial species** | **No of isolates** | **Bacterial species** | **No of isolates** |
| *Escherichia coli* | 42 | *Klebsiella pneumoniae* | 7 |
| *Shigella sonnei* | 3 | *Escherichia coli* | 4 |
| *Shigella flexneri* | 4 | *Acinetobacter baumannii* | 4 |
| *Salmonella typhimurium* | 3 | Total | 15 |
| *Salmonella Senftenberg* | 2 |  |  |
| *Salmonella Albert* | 1 |  |  |
| *Salmonella Concord* | 1 |  |  |
| *Salmonella Corvallis* | 1 |  |  |
| *Salmonella Cubana* | 1 | **ATCC (Target (Sanger) sequencing)** | |
| *Salmonella Stanley* | 1 | **Bacterial species** | **No of isolates** |
| *Salmonella Heidelberg* | 1 | *Escherichia coli* | 6 |
| *Campylobacter jejuni* | 5 | *Klebsiella pneumoniae* | 3 |
| *Campylobacter coli* | 5 | *Pseudomonas aeruginosa* | 1 |
| *Klebsiella pneumoniae* | 51 | *Enterococcus faecium* | 1 |
| *Klebsiella oxytoca* | 3 | *Enterococcus faecalis* | 2 |
| *Klebsiella ozaenae* | 2 | *Staphylococcus aureus* | 4 |
| *Acinetobacter baumannii* | 14 | *Staphylococcus epidermidis* | 1 |
| *Pseudomonas aeruginosa* | 12 | *Staphylococcus saprophytigus* | 1 |
| *Enterobacter cloacae* | 16 | *Acinetobacter baumannii* | 1 |
| *Enterobacter aerogenes* | 6 | Total | 20 |
| *Citrobacter freundii* | 4 |  |  |
| *Citrobacter koseri* | 2 |  |  |
| *Citrobacter species* | 1 |  |  |
| *Serratia marcescens* | 9 |  |  |
| *Proteus mirabilis* | 5 |  |  |
| *Providencia stuartii* | 1 |  |  |
| *Providencia rettgeri* | 1 |  |  |
| *Morganella morganii* | 2 |  |  |
| *Raoultella Ornithinolytica* | 1 |  |  |
| *Kluyvera ascorbata* | 1 |  |  |
| Total | 201 |  |  |
