# Supplementary material for: Genotypic antimicrobial resistance assays for use on E. coli isolates and stool specimens
Source: PLoS One. 2019 May 10;14(5):e0216747. doi: 10.1371/journal.pone.0216747 (PMC6510447; doi:10.1371/journal.pone.0216747)
Supplement: S4 Table — (DOCX) [file pone.0216747.s004.docx]

**S4 Table. Primer and probe sequences of the 42 duplex and 1 singleplex PCR reactions.**

| **Duplex Assay #** | **Target 1** | **Sequences (5’-3’)^a^** | **References for S4 table** | **Target 2** | **Sequences (5’-3’)^a^** | **Reference for S4 table** |
| --- | --- | --- | --- | --- | --- | --- |
| 1 | TEM 104E | F-CGGTCGCCGCATACACTAT | This study | TEM 104K | F-CGGTCGCCGCATACACTAT | This study |
|  |  | R-TCATGCCATCCGTAAGATGC |  |  | R-TCATGCCATCCGTAAGATGC |  |
|  |  | FAM-ACTTGGTTGAGTACTCAC-MGB |  |  | VIC-ACTTGGTTAAGTACTCACC-MGB | [1] |
| 2 | TEM 164R | F-TTCTGACAACGATCGGAGGA | This study | TEM 164SC | F-TTCTGACAACGATCGGAGGA | This study |
|  |  | R-CGTCGTTTGGTATGGCTTCA |  |  | R-CGTCGTTTGGTATGGCTTCA |  |
|  |  | FAM-TTCCCAACGATCAAG-MGB |  |  | VIC-TTCCCAACWATCAAGGC-MGB |  |
| 3 | PhHV (*gB*) | F-GGGCGAATCACAGATTGAATC | [2] | TEM 238S | F-GCTGGCTGGTTTATTGCTGATA | This study |
|  |  | R-GCGGTTCCAAACGTACCAA |  |  | R-GCCCCAGTGCTGCAATG |  |
|  |  | FAM-TATGTGTCCGCCACCATCT-MGB |  |  | VIC-ATCTGGAGCCAGTGAG-MGB |  |
| 4 | SHV | F-TCCCATGATGAGCACCTTTAAA | [3] | SHV 238-240SE-SK | F-GTTGATCCGYTCCGTGCT | This study |
|  |  | R-TCCTGCTGGCGATAGTGGAT |  |  | R-GCTTTGTTATKCGGGCCAAG |  |
|  |  | FAM-TGCCGGTGACGAACAGCTGGAG-MGB |  |  | VIC-CGGAGCTAGCRARC-MGB |  |
| 5 | CTX-M1 | F-CCGTCACGCTGTTRTTAGGA | This study | CTX-M8-M25 | F-ATRACACSTTCCGGCTCGAT | [1] |
|  |  | R-AATGCCACMCCCAGYCKKCC |  |  | R-GCTAAYGGCGTGGTGGTATC |  |
|  |  | FAM-CAGCAAAAACTTGCCGRATT-MGB |  |  | VIC-TCAACACCGCGATCCCCG-MGB |  |
| 6 | CTX-M2-M74 | F-GCGCAGACCCTGAAAAAYCT | This study | CTX-M9 | F-GCTTTATGCGCAGACGARTG | This study |
|  |  | R-TGYGCSCGCTGRGTTTCC |  |  | R-ATCACCGCGATAAAGCACCT |  |
|  |  | FAM-ACSCTGGGYAAAGCGC-MGB |  |  | VIC-TCGATACCRMAGATAATACGC-MGB |  |
| 7 | PER | F-CCCACTGTTAAAAGAGCAAATTGA | This study | VEB | F-GTAGCAATATTCAACAGCAATGAGAAG | This study |
|  |  | R-CGTCAGGCCCCCACACT |  |  | R-CGGCTAAAGCAATCGGAAATT |  |
|  |  | FAM-AAAAAGCCACTGTAGGCGTTG-MGB |  |  | VIC-TAACGACTTCCATTWCCCGA-MGB |  |
| 8 | CMY1-MOX | F-GCAACAACGACAATCCATCC | This study | FOX | F-CTGTGGACGGCATTATCCAG | This study |
|  |  | R-AARTAGTGGGCCTTGCCATC |  |  | R-TAGTGGGCYTTGCCATCTTT |  |
|  |  | FAM-CTGCTCAAGGAGCACAGGAT-MGB |  |  | VIC-ATGCTCAAGGAGTATCG-MGB |  |
| 9 | CMY2-LAT | F-GKHGGATTYGGCAGCTACGT | This study | ACT-MIR | F-YGGRTTTGGCAGCTACKT | This study |
|  |  | R-GYTYTTRTTTGCCARCATYA |  |  | R-GCTTKTATTYGCSAGCATYACA |  |
|  |  | FAM-TCRTTCCRGAAAAAAA-MGB |  |  | VIC-CTGCTTTTCRGGAATAAA-MGB |  |
| 10^b^ | DHA | F-GYGCYTATGTSGCCTTTATTC | This study |  |  |  |
|  |  | R-TCTTTCGGTATTCGGGTAGTTTTT |  |  |  |  |
|  |  | FAM-CAGGTGGCGATTGTGAT-MGB |  |  |  |  |
| 11 | KPC | F-GGCCGCCGTGCAATAC | [4] | GES | F-TGGCTGATCGGAAACCAAA | This study |
|  |  | R-GCCGCCCAACTCCTTCA |  |  | R-GGCGCAGGYACCAGTTTTCTC |  |
|  |  | FAM-TGATAACGCCGCCGCCAATTTGT-MGB |  |  | VIC-CTACGAGCGGGTTTTCC-MGB |  |
| 12 | NDM | F-ATATCACCGTTGGGATCGAC | [1] | VIM | F-TSTACCCRTCCAATGGTCTC | [1] Modify |
|  |  | R-TAGTGCTCAGTGTCGGCATC |  |  | R-AGAAGKGCCRCTGTGTTTTT |  |
|  |  | FAM-AAGGACAGCAAGGCCAAGTCG-MGB |  |  | VIC-TGTCCGTGATGGYGATGAGTTG-MGB |  |
| 13 | IMP | F-AGTRGTTTGGYTRCCTRAAA | This study | OXA-48 | F-GCAAAGGAATGGCAAGAAAA | [1] |
|  |  | R-TKTGGCCAWGCTTYWAHATTTGC |  |  | R-CACAACTACGCCCTGTGATTT |  |
|  |  | FAM-TTYGGTGGTTGYTTTRTTAA-MGB |  |  | VIC-AGTTGGAATGCTCACTTTACTG-MGB |  |
| 14 | OXA-1 | F-CAGCGCCAGTGCATCAAC | This study | OXA-9 | F-CCGTGGCTTCTGATGAGGTT | This study |
|  |  | R-TTCAGCGTTTGTGGATRCAT |  |  | R-ACACGACGGGCACATTCTC |  |
|  |  | FAM-ATATCTCTACTGTTGCATCTC-MGB |  |  | VIC-CTTAAATGCACCATCATC-MGB |  |
| 15 | QnrA | F-GGATTTGAGYGACAGYCGTTTT | This study | QnrS | F-TTGCTCAGCMTTTATTWCWGGATGT | This study |
|  |  | R-CAATGAAACTGCAATCCTCGAA |  |  | R-CAGCGATTTTCAWACARCTCACA |  |
|  |  | FAM-CCGCTGCCGCTTYTATCA-MGB |  |  | VIC-TATGCCAATATGGAGMGGGT-MGB |  |
| 16 | QnrB1 | F-GYGGCGAGTTTWCGACTTT | This study | QnrB4 | F-ACVCGSACCTGGTTTTGYAG | This study |
|  |  | R-TRGTCAGATCGCAATGTGTG |  |  | R-GTACCCATCCAGCGGTTTTC |  |
|  |  | FAM-ACTGGCGAGCAGSRAACTT-MGB |  |  | VIC-CTKGAAAAGTGCGARYTGT-MGB |  |
| 17 | aac(6’)-lb-104W | F-TATGCCCAGTCGTACGTTGC | This study | aac(6’)-lb-104R | F-TATGCCCAGTCGTACGTTGC | This study |
|  |  | R-GTTGTGATGCATTCGCCAGT |  |  | R-GTTGTGATGCATTCGCCAGT |  |
|  |  | FAM-TCCCACCATCCGTCC-MGB |  |  | VIC-TCCCACCKTCCGTCC-MGB |  |
| 18 | gyrA87G-ESh^c^ | F-TGGTGACGTAATCGGTAAATACCA | This study | aac(6’)-lb-181Y | F-CGATCCGATGCTACGAGAAAG | This study |
|  |  | R-CCGAAGTTACCCTGACCGTCT |  |  | R-CCTGGCGTGTTTGAACCAT |  |
|  |  | FAM-GTYTATGGCACGATC-MGB |  |  | VIC-CACCCCATATGGTCC-MGB |  |
| 19 | QepA | F-TGTTCGGCGTCTACATCTTCA | This study | gyrA87G-Sal^d^ | F-TGGTGACGTAATCGGTAAATACCA | This study |
|  |  | R-GAACCGATGACGAAGCACAG |  |  | R-ATCAGTTCGTGGGCGATTTT |  |
|  |  | FAM-GACGCAGTACCTGCAGCTC-MGB |  |  | VIC-GTGTATGGCACCATC-MGB |  |
| 20 | gyrA83S-Sal^d^ | F-TGGTGACGTAATCGGTAAATACCA | This study | gyrA83FY-Sal^d^ | F-TGGTGACGTAATCGGTAAATACCA | This study |
|  |  | R-ATCAGTTCGTGGGCGATTTT |  |  | R-ATCAGTTCGTGGGCGATTTT |  |
|  |  | FAM-CACTGCGGAATC-MGB |  |  | VIC-TGCGWAATCGCC-MGB |  |
| 21 | gyrA87D-Sal^d^ | F-TGGTGACGTAATCGGTAAATACCA | This study | gyrA87NY-Sal^d^ | F-TGGTGACGTAATCGGTAAATACCA | This study |
|  |  | R-ATCAGTTCGTGGGCGATTTT |  |  | R-ATCAGTTCGTGGGCGATTTT |  |
|  |  | FAM-AGTGTATGACACCATC-MGB |  |  | VIC-GCAGTGTATWACACCAT-MGB |  |
| 22 | gyrA83S-ESh^c^ | F-TGGTGACGTAATCGGTAAATACCA | This study | gyrA83L-ESh^c^ | F-TGGTGACGTAATCGGTAAATACCA | This study |
|  |  | R-CCGAAGTTACCCTGACCGTCT |  |  | R-CCGAAGTTACCCTGACCGTCT |  |
|  |  | FAM-ARACYGCCGAGTCA-MGB |  |  | VIC-ACYGCCAAGTCAC-MGB |  |
| 23 | gyrA87D-ESh^c^ | F-TGGTGACGTAATCGGTAAATACCA | This study | gyrA87NY-ESh^c^ | F-TGGTGACGTAATCGGTAAATACCA | This study |
|  |  | R-CCGAAGTTACCCTGACCGTCT |  |  | R-CCGAAGTTACCCTGACCGTCT |  |
|  |  | FAM-CGATCGTGTCATARAC-MGB |  |  | VIC-CGATCGTGTWATARA-MGB |  |
| 24 | parC80S-Sal^d^ | F-GACGTACTGGGTAAGTATCACCCG | This study | parC80I-Sal^d^ | F-GACGTACTGGGTAAGTATCACCCG | This study |
|  |  | R-ATCGCCGCGAATGACTTC |  |  | R-ATCGCCGCGAATGACTTC |  |
|  |  | FAM-CAGGCGCTGTCGC-MGB |  |  | VIC-CAGGCGATGTCG-MGB |  |
| 25 | parC80S-ESh^c^ | F-CTGAACTGGGCCTGAATGC | This study | parC80I-ESh^c^ | F-CTGAACTGGGCCTGAATGC | This study |
|  |  | R-ATTGCCGCGAACGATTTC |  |  | R-ATTGCCGCGAACGATTTC |  |
|  |  | FAM-CAGGCRCTATCGC-MGB |  |  | VIC-CAGGCRATATCG-MGB |  |
| 26 | gyrA86T-Cj^e^ | F-GCCCGTATAGTGGGTGCTGT | This study | gyrA86I-Cj^e^ | F-GCCCGTATAGTGGGTGCTGT | This study |
|  |  | R-TCTTGAGCCATTCTAACCAAAGC |  |  | R-TCTTGAGCCATTCTAACCAAAGC |  |
|  |  | FAM-ATAAACTGCTGTATCTC-MGB |  |  | VIC-AACTGCTATATCTCC-MGB |  |
| 27 | gyrA86T-Cc^f^ | F-TCTGCTCGTATAGTAGGGGATGTT | This study | gyrA86I-Cc^f^ | F-TCTGCTCGTATAGTAGGGGATGTT | This study |
|  |  | R-GCATAGAGAAATCTTGTGCCATTC |  |  | R-GCATAGAGAAATCTTGTGCCATTC |  |
|  |  | FAM-GTAAACAGCAGTATCG-MGB |  |  | VIC-ACAGCAATATCGC-MGB |  |
| 28 | 23S-2075A-Cp^g^ | F-GATCCAGTGAAATTGTAGTGGAGGT | [5] | 23S-2075G-Cp^g^ | F-GATCCAGTGAAATTGTAGTGGAGGT | [5] |
|  |  | R-GGCTCATATACAACTGGCGTCATA | This study |  | R-GGCTCATATACAACTGGCGTCATA | This study |
|  |  | FAM-GACGGAAAGACC-MGB | This study |  | VIC-GACGGAGAGACCC-MGB | This study |
| 29 | ermB | F-CGTACCTTGGATATTCACCG | [6] | mphA | F-GTGCTGGCAATGCTCAAGAA | This study |
|  |  | R-GTAAACAGTTGACGATATTCTCG |  |  | R-TGACCATCGCAGTCGAGTCT |  |
|  |  | FAM-TGCACACTCAAGTCTCGATTCAGC-MGB |  |  | VIC-AGCTCGTTGCCTATCCCAT-MGB |  |
| 30 | armA | F-TGTGGCTTCAATCCATTAGCTTTAT | This study | rmtB | F-TCCCCCAAACAGRCCGTAGA | This study |
|  |  | R-TCTCAGCTCTATCAATATCGTATGCA |  |  | R-ATATGCCCCGCAAATTCCAT |  |
|  |  | FAM-CCAATGGAATGAAAATGAAAAAAT-MGB |  |  | VIC-CTGCACGCACCCGGC-MGB |  |
| 31 | aacC1 | F-GCGCTTGGTGCTTATGTGATC | This study | aacC2 | F-TATGAGATGCCGATGCTTGG | This study |
|  |  | R-TTCTTCCCGTATGCCCAACT |  |  | R-CATCCGGCTTTCCTTCGATA |  |
|  |  | FAM-ACGTGCAAGCAGATTAC-MGB |  |  | VIC-AAACGGCATTCTCGATTG-MGB |  |
| 32 | aacC4 | F-CATGAACTCGATGGGCAGGTA | This study | aadB | F-TGTAACACGCAAGCACGATGA | This study |
|  |  | R-GGGAACCTTTGCCATCAACTC |  |  | R-CCGCCGAGCATTTCAACTAT |  |
|  |  | FAM-ACACGATGCCAACACGACG-MGB |  |  | VIC-ATTGATCTGACGTTTCCC-MGB |  |
| 33 | aphA1 | F-GCTGGCCTGTTGAACAAGTCT | This study | aadA1-2-17 | F-ATYATBCCGTGGCGTTATCC | This study |
|  |  | R-ATCACCATGAGTGACGACTGAATC |  |  | R-RTGGCTGGCTCGAAGATACC |  |
|  |  | FAM-CATAAGCTTTTGCCATTCT-MGB |  |  | VIC-CCATTCTCCAAATTGCAG-MGB |  |
| 34 | dfrA1 | F-TCGGGAATGGCCCTGATAT | This study | dfrA12 | F-CCGAACCGTCACACATTGG | This study |
|  |  | R-GTCCAACCAACAGCCATTGG |  |  | R-TGCGACAGCGTTGAAACAAC |  |
|  |  | FAM-CCATGGAGTGCCAAAGGT-MGB |  |  | VIC-AATCTCACGCCAAGCTA-MGB |  |
| 35 | dfrA5-14 | F-TTCTTYCCGARTATTCCMAATACC | This study | dfrA17 | F-GCAGTGTCAGAAAATGGCGTAA | This study |
|  |  | R-CCCTTTTKCCAAATTTGATAGC |  |  | R-CCGACAAGGAGCCATTGATT |  |
|  |  | FAM-CGAAGTTGTTTTTGAGCAACA-MGB |  |  | VIC-TAGTGGTCCTGATATCCCGTG-MGB |  |
| 36 | sul1 | F-CGTGCTGTCGAACCTTCAAA | This study | sul2 | F-CGTGGTGTGGCCTATCTCAA | This study |
|  |  | R-CCTTTACAGGAAGGCCAACG |  |  | R-ACGACGAGTTTGGCAGATGA |  |
|  |  | FAM-AGAAGGATTTCCGCGACAC-MGB |  |  | VIC-GATATTCGCGGTTTTCCAGA-MGB |  |
| 37 | sul3 | F-TTGGTTGAAGATGGAGCAGATG | This study | Bacterial 16S | F-TGGAGCATGTGGTTTAATTCGA | [7] |
|  |  | R-TCCACAACGCCCACTTCAG |  |  | R-TGCGGGACTTAACCCAACA |  |
|  |  | FAM-ATTGATTTGGGAGCCGCT-MGB |  |  | VIC-CGAGCTGACGACARCCRTGCA-MGB |  |
| 38 | tetA | F-CGACGGCACAGGCTACATC | This study | tetB | F-CCGTTTGCTTTCAGGGATCA | This study |
|  |  | R-CCTGGACAACATTGCTTGCA |  |  | R-CACTTCACGCGTTGAGAAGCT |  |
|  |  | FAM-TGGCGTTCCCGATCAT-MGB |  |  | VIC-TCATTGCCGATACCACC-MGB |  |
| 39 | catA1 | F-GCCAATCCCTGGGTGAGTTT | This study | catB3 | F-AAAAGATGTGGAGCCTTACGC | This study |
|  |  | R-ACCTTGTCGCCTTGCGTATAA |  |  | R-GCCGCTTTGATCTTCTCCAG |  |
|  |  | FAM-TTTAAACGTGGCCAATATG-MGB |  |  | VIC-AGATGGAGTGGTGGAATTGG-MGB |  |
| 40 | cmlA | F-ACTCCCCGTTAAGTGCCTGA | This study | floR | F-TTTTGGTCCGCTCTCAGACA | This study |
|  |  | R-GGCGCAATGGAGAAAAAGAC |  |  | R-CCAGAGACGCAATGACGAAA |  |
|  |  | FAM-TACACGTTGTGTTACGCC-MGB |  |  | VIC-CGCCCGCAAGTAGAATT-MGB |  |
| 41 | mcr-1 | F-GATCGCTGTCGTGCTCTTTG | This study | mcr-2 | F-TGACATCACAKCACTCTTGGTATCG | This study |
|  |  | R-ACCGCGCCCATGATTAATAG |  |  | R-TGTYGCYGCCAAAAATAACG |  |
|  |  | FAM-CGATGCTACTGATCACCACG-MGB |  |  | VIC-CTTTTGTRCTGATGGGTTTGGT-MGB |  |
| 42 | *E.coli-Shigella* spp. (*uidA*) | F-GAGCATCAGGGTGGCTATACG | [8] | *Shigella* spp. (*ipaH*) | F-CCTTTTCCGCGTTCCTTGA | [9] |
|  |  | R-ATAGTCTGCCAGTTCAGTTC |  |  | R-CGGAATCCGGAGGTATTGC |  |
|  |  | FAM-TACGGCGTGACATCGGCTTCAAATG-MGB |  |  | VIC-CGCCTTTCCGATACCGTCTCTGCA-MGB |  |
| 43 | *Salmonella* spp. (*ttr*) | F-CTCACCAGGAGATTACAACATGG | [10] | *C. jejuni-coli* (*cadF*) | F-CWGCTAAACCATARAAATAAAATTTCTCAC | [11] |
|  |  | R-AGCTCAGACCAAAAGTGACCATC |  |  | R-YTTTGAAGGTAATTTAGATATGGATAATCG |  |
|  |  | FAM-CACCGACGGCGAGACCGACTTT-MGB |  |  | VIC-CATTTTGAYGATTTTTGGCTTGA-MGB |  |

^a^ F; forward primer, R; reverse primer, MGB; minor groove binding

^b^This assay is singleplex

^c^ESh ; *E.coli-Shigella* spp., ^d^ Sal ; *Salmonella* spp., ^e^ Cj ; *C. jejuni*, ^f^ Cc ; *C. coli*, ^g^ Cp ; *Campylobacter* spp.

**References**

1. Chavda KD, Satlin MJ, Chen L, Manca C, Jenkins SG, Walsh TJ, et al. Evaluation of a Multiplex PCR Assay To Rapidly Detect Enterobacteriaceae with a Broad Range of beta-Lactamases Directly from Perianal Swabs. Antimicrobial agents and chemotherapy. 2016;60(11):6957-61. Epub 2016/09/08. doi: 10.1128/AAC.01458-16. PubMed PMID: 27600053; PubMed Central PMCID: PMC5075117.

2. Liu J, Gratz J, Amour C, Kibiki G, Becker S, Janaki L, et al. A laboratory-developed TaqMan Array Card for simultaneous detection of 19 enteropathogens. Journal of clinical microbiology. 2013;51(2):472-80. Epub 2012/11/24. doi: 10.1128/JCM.02658-12. PubMed PMID: 23175269; PubMed Central PMCID: PMC3553916.

3. Roschanski N, Fischer J, Guerra B, Roesler U. Development of a multiplex real-time PCR for the rapid detection of the predominant beta-lactamase genes CTX-M, SHV, TEM and CIT-type AmpCs in Enterobacteriaceae. PloS one. 2014;9(7):e100956. Epub 2014/07/18. doi: 10.1371/journal.pone.0100956. PubMed PMID: 25033234; PubMed Central PMCID: PMC4102473.

4. Lee TD, Adie K, McNabb A, Purych D, Mannan K, Azana R, et al. Rapid Detection of KPC, NDM, and OXA-48-Like Carbapenemases by Real-Time PCR from Rectal Swab Surveillance Samples. Journal of clinical microbiology. 2015;53(8):2731-3. Epub 2015/05/29. doi: 10.1128/JCM.01237-15. PubMed PMID: 26019195; PubMed Central PMCID: PMC4508430.

5. Hao H, Liu J, Kuang X, Dai M, Cheng G, Wang X, et al. Identification of Campylobacter jejuni and determination of point mutations associated with macrolide resistance using a multiplex TaqMan MGB real-time PCR. Journal of applied microbiology. 2015;118(6):1418-25. Epub 2015/03/15. doi: 10.1111/jam.12793. PubMed PMID: 25766481.

6. Kumari N, Navaratnam P, Sekaran SD. Detection of pbp2b and ermB genes in clinical isolates of Streptococcus pneumoniae. Journal of infection in developing countries. 2008;2(3):193-9. Epub 2008/01/01. PubMed PMID: 19738350.

7. Sinsimer D, Leekha S, Park S, Marras SA, Koreen L, Willey B, et al. Use of a multiplex molecular beacon platform for rapid detection of methicillin and vancomycin resistance in Staphylococcus aureus. Journal of clinical microbiology. 2005;43(9):4585-91. Epub 2005/09/08. doi: 10.1128/JCM.43.9.4585-4591.2005. PubMed PMID: 16145111; PubMed Central PMCID: PMC1234154.

8. Diaz MH, Waller JL, Napoliello RA, Islam MS, Wolff BJ, Burken DJ, et al. Optimization of Multiple Pathogen Detection Using the TaqMan Array Card: Application for a Population-Based Study of Neonatal Infection. PloS one. 2013;8(6):e66183. Epub 2013/06/28. doi: 10.1371/journal.pone.0066183. PubMed PMID: 23805203; PubMed Central PMCID: PMC3689704.

9. Vu DT, Sethabutr O, Von Seidlein L, Tran VT, Do GC, Bui TC, et al. Detection of Shigella by a PCR assay targeting the ipaH gene suggests increased prevalence of shigellosis in Nha Trang, Vietnam. Journal of clinical microbiology. 2004;42(5):2031-5. Epub 2004/05/08. PubMed PMID: 15131166; PubMed Central PMCID: PMC404673.

10. Malorny B, Cook N, D'Agostino M, De Medici D, Croci L, Abdulmawjood A, et al. Multicenter validation of PCR-based method for detection of Salmonella in chicken and pig samples. Journal of AOAC International. 2004;87(4):861-6. Epub 2004/08/07. PubMed PMID: 15295881.

11. Cunningham SA, Sloan LM, Nyre LM, Vetter EA, Mandrekar J, Patel R. Three-hour molecular detection of Campylobacter, Salmonella, Yersinia, and Shigella species in feces with accuracy as high as that of culture. Journal of clinical microbiology. 2010;48(8):2929-33. Epub 2010/06/04. doi: 10.1128/JCM.00339-10. PubMed PMID: 20519461; PubMed Central PMCID: PMC2916566.
