# Supplementary material for: Genotypic antimicrobial resistance assays for use on E. coli isolates and stool specimens
Source: PLoS One. 2019 May 10;14(5):e0216747. doi: 10.1371/journal.pone.0216747 (PMC6510447; doi:10.1371/journal.pone.0216747)
Supplement: S5 Table — (DOCX) [file pone.0216747.s005.docx]

**S5 Table.** **Sequencing primers.**

| **Gene targets** | **Sequences (5’-3’)^a^** | **Product**  **size (bp)** | **Gene targets** | **Sequences (5’-3’)^a^** | **Product**  **size (bp)** |
| --- | --- | --- | --- | --- | --- |
| TEM | For-TCGARCTGGATCTCAACAGC | 707 | mphA | For-CAATGAGCTTGGGCTCGACT | 646 |
|  | Rev-GGCACCTATCTCAGCGATCTG |  |  | Rev-GAGCCCCTCTTYACCAAAGA |  |
| SHV | For-CMGCGTAGGCATGATAGAAA | 668 | armA | For-CGAAAACCTTGATTCAGATATTGTTG | 614 |
|  | Rev-CCCGCAGATAAATCACCACR |  |  | Rev-CCATTCCCTTCTCCTTTCCA |  |
| CTX-M | For-GATGARCGYTTTSCVATGT | 610 | rmtB | For-GAACATCAACGATGCCCTCA | 654 |
|  | Rev-GYTGGGTRAARTARGTSACCA |  |  | Rev-TAGTTCGCCTCCATGCCTTT |  |
| CTX-M1 | For-CCGTCACGCTGTTGTTAGGA | 613 | aacC1 | For-GGCAGTCGCCCTAAAACAAA | 469 |
|  | Rev-CCGGTGGTATTGCCTTTCAT |  |  | Rev-GCATCACTTCTTCCCGTATGC |  |
| CTX-M9 | For-GCTTTATGCGCAGACGAGTG | 669 | aacC2 | For-GACCGATCACCCTACGAGGA | 676 |
|  | Rev-TCATTGGTGGTGCCGTAGTC |  |  | Rev-AACCKGAAGGCTCGCAAGAG |  |
| PER | For-TCCCCDYTGTTAAAAGRGCARA | 680 | aacC4 | For-TCGGTCAGCTTCTCAACCTT | 605 |
|  | Rev-TTRGTSGCCGCMGTTTTYCC |  |  | Rev-ACCGACTGGACCTTCCTTCT |  |
| VEB | For-TTCAAATGCTCAARCTGACAACTT | 610 | aadB | For-GGACACAACGCAGGTCACAT | 504 |
|  | Rev-TCGGTTACTTCCTGYTGTTGTTTC |  |  | Rev-GCACGCAAGACCTCAACCTT |  |
| CMY1-MOX | For-GCTGCTCAAGGAGCACAGG | 634 | aphA1 | For-CGAGGCMGCGATTAAATTCC | 708 |
|  | Rev-CGCCGARCTGGTYTTGAT |  |  | Rev-GCCGTTTCTGTAATGAAGGAGAA |  |
| FOX | For-CATTATCCAGCCGATGCTCA | 591 | aadA1 | For-GCCTGAAGCCAYACAGYGAT | 657 |
|  | Rev-CGGAYRGGCTTMTCTTCCTT |  |  | Rev-AGTCGGCAGCGACATCCT |  |
| CMY2-LAT | For-YKCTAACTCCAGCATTGGTCT | 580 | dfrA1 | For-TGGTAGCTATATCGAAGAATGGA | 454 |
|  | Rev--GYTYTTRTTTGCCARCATYA |  |  | Rev-CCCTTTTGCCAGATTTGGTAACTAT |  |
| ACT-MIR | For-CCAGYATCGGNCTKTTTGG | 610 | dfrA12 | For-TTATCTCGTTGCTGCGATGG | 417 |
|  | Rev-ATABGGTAWGCCGCYTCMAC |  |  | Rev-TCGGTTGAGACAAGCTCGAA |  |
| DHA | For-GGGMDAKATGCGTCTGTATG | 650 | dfrA5-14 | For-ATRGCTGCRAAAGCGAAAAA | 456 |
|  | Rev-ATTCCAGYGCACTCAAAATAGCC |  |  | Rev-ACCCTTTTKCCAAATTTGATAGC |  |
| KPC | For-TGTAAGTTACCGCGCTGAGG | 622 | dfrA17 | For-GAAAATATCATTGATTTCTGCAGTGTC | 465 |
|  | Rev-GGTGTAGACGGCCAACACAA |  |  | Rev-TTTTTCCAAATCTGGTATGTATAATTT |  |
| GES | For-TGTGCTCAACGTTCAAGTTTCC | 602 | sul1 | For-CACCGAGGACTCCTTCTTCG | 644 |
|  | Rev-TAGTTTCGGGGCCGTTGTAT |  |  | Rev-CCTTTACAGGAAGGCCAACG |  |
| NDM | For-AATGGAAACTGGCGACCAAC | 687 | sul2 | For-TCATTTTCGGCATCGTCAAC | 693 |
|  | Rev-YGGGCCGTATGAGTGATTG |  |  | Rev-CAAGCTCTGCAGCGAGTGTC |  |
| VIM | For-CAGATTGMYGATGGTGTTTG | 653 | sul3 | For-TCCGATGGAGGACTTTATTTAGA | 700 |
|  | Rev-RCGACTGAGCGATTTKTGTG |  |  | Rev-CATCATGGGTGCGGAGATAA |  |
| IMP | For-RCTTGAHRAVGRYGTTTWTSTTC | 617 | tetA | For-GCTTCATGAGCGCCTGTTT | 706 |
|  | Rev-RYCCYTTWASVGCHTGYTCY |  |  | Rev-CACCCGTTCCACGTTGTTAT |  |
| OXA-48 | For-GCCTTATCGGCTGTGTTTTTG | 655 | tetB | For-TGGCGTATTGCTTGCACTTT | 680 |
|  | Rev-CCCACCAGCCAATCTTAGGT |  |  | Rev-TGGCTATTCTTCCTGCCACA |  |
| OXA-1 | For-CGCCAGTGCATCAACAGATA | 702 | catA1 | For-TCCCAATGGCATCGTAAAGA | 610 |
|  | Rev-TTCGACCCCAAGTTTCCTGT |  |  | Rev-CCTGCCACTCATCGCAGTA |  |
| OXA-9 | For-TGCTGCTGCATATGTTGGTG | 726 | catB3 | For-GATAGCCCCTTCAAAGGCAAG | 551 |
|  | Rev-CTATTTCCAATCGGGCGAAA |  |  | Rev-GCCGCTTTGATCTTCTCCAG |  |
| QnrA | For-AAAGTTTTTCARCAAGAGGATTTC | 640 | cmlA | For-GCAGGTCGCGAGGAAAGTAA | 630 |
|  | Rev-AATCCGGMAGCACTATKAYYC |  |  | Rev-TCCAGCAACATGGTCGAATC |  |
| QnrS | For-GAYTTTCGACGWGCTAACTTGC | 550 | floR | For-CCTTTCGACATCCTCGCTTC | 699 |
|  | Rev-CAACAATACCCARYGCTTCGAG |  |  | Rev-GGCAAAGCTGAATCCGATCT |  |
| QnrB1 | For-TTTTTWAYTGYGATTTTTCAGGTG | 501 | mcr-1 | For-CTATCCCATCGCGGACAATC | 722 |
|  | Rev-TGTAAATCAACVCBMCGAAT |  |  | Rev-CGCACGATGTGACATTGCTA |  |
| QnrB4 | For-AAAAATTGACAGAAACMGVTTCA | 516 | mcr-2 | For-GACATCACAKCACTCTTGGTATCG | 728 |
|  | Rev-TCYGARTTGGTCARRTCACAGT |  |  | Rev-AAGCTGYGGGAAAGTCTCAC |  |
| aac(6’)-lb | For-TTGCGATGCTCTATGRGTGGCTA | 474 | gyrA-*C.coli* | For-TGCACTTCCTGACGCTAGAGA | 513 |
|  | Rev-CMTGGCGTGTTTGAACCAY |  |  | Rev-GCATCCTTATGATCAAGCAAA |  |
| QepA | For-GATCTACGGGCTCAAGCAGT | 500 | parC-*Salmonella* spp. | For-ATCGTGCGTTGCCGTTTATT | 588 |
|  | Rev-TGGTGATGATGATCTCGTTGC |  |  | Rev-GGGTAATGATCTCCGCTTCG |  |
| gyrA-*Salmonella* spp. | For-GGATTATGCGATGTCGGTCA | 506 | parC-*E.coli* | For-TTGCCGTTTATTGGTGATGG | 513 |
|  | Rev-TCACTTCCGTCAGGTTGTGC |  |  | Rev-GGTTTTCGGCTGGTCGATTA |  |
| gyrA-*E.coli* | For-CACCGGTCAACATTGAGGAA | 503 | 23S-*Campylobacter* spp. | For-CAAAGAGTCCCTCCCGACTG | 551 |
|  | Rev-TACGGCGATACCGGAAGAAC |  |  | Rev-CAACCGTTCTGAGCCAACCT |  |
| gyrA-*C.jejuni* | For-TTTGCCTGACGCAAGAGATG | 564 | ermB | For-CGAGTGAAAARGTACTCAACCAAA | 620 |
|  | Rev-CCACCTGTTGGAAAATCTGGA |  |  | Rev-TTTGGCGTRTTTCATTGCTT |  |

^a^ The primers were designed in this study, For; forward, Rev; reverse
