# Supplementary material for: Genotypic antimicrobial resistance assays for use on E. coli isolates and stool specimens
Source: PLoS One. 2019 May 10;14(5):e0216747. doi: 10.1371/journal.pone.0216747 (PMC6510447; doi:10.1371/journal.pone.0216747)
Supplement: S7 Table — (DOCX) [file pone.0216747.s007.docx]

**S7 Table.** **Antimicrobial agent classes and gene targets included in AMR-TAC.**

| **Antimicrobial classes** | **Resistance mechanisms** | **Target genes/ Enzymes** | **PCR target** | **References for S7 table** |
| --- | --- | --- | --- | --- |
| Penicillins | Antibiotic hydrolyzation | class A β-lactamase | TEM, SHV | [1, 2] |
| Cephalosporins (3th and 4th generation) | Antibiotic hydrolyzation | class A β-lactamase | CTX-M, TEM-ESBL, SHV-ESBL, PER, VEB, GES | [3-10] |
|  |  | plasmid mediated AmpC β-lactamase | CMY, DHA, FOX, MOX, ACT, MIR, LAT |  |
| Carbapenems | Antibiotic hydrolyzation | class A β-lactamase | KPC | [6, 11-16] |
|  |  | class B β-lactamase | NDM, IMP, VIM |  |
|  |  | class D β-lactamase | OXA-48 |  |
| Quinolones | Antibiotic target modification | *gyrA* | S83L-Y-F, D87N-Y-G, T86I | [17-23] |
|  |  | *parC* | S80I |  |
|  | Antibiotic target protection | *qnr* | *qnrS, qnrB, qnrA* |  |
|  | Antibiotic modification | acetyltransferase | *aac(6’)-lb-cr* |  |
|  | Efflux pump | *qepA* | *qepA* |  |
| Macrolides | Antibiotic modification | phosphotransferase | *mphA* | [3, 24-28] |
|  | Antibiotic target modification | rRNA methylase | *ermB* |  |
|  |  | 23S mutation | A2075G |  |
| Aminoglycosides | Antibiotic modification | acetyltransferase | *aacC1, aacC2, aacC4, aac(6’)-Ib* | [29-32] |
|  |  | phosphotransferase | *aphA1* |  |
|  |  | nucleotidyltransferase | *aadA, aadB* |  |
|  | Antibiotic target modification | 16S methylase | *armA, rmtB* |  |
| Polymyxins | Antibiotic target modification | phosphoethanolamine transferase | *mcr-1, mcr-2* | [33-36] |
| Folate partway inhibitors | Acquisition of drug-resistant target enzymes | dihydropteroate synthase | *sul1, sul2, sul3* | [37-42] |
|  |  | dihydrofolate reductase | *dfrA1, dfrA5, dfrA12, dfrA14, dfrA17* |  |
| Tetracyclines | Efflux pumps | *tetA, tetB* | *tetA, tetB* | [43, 44] |
| Phenicols | Efflux pumps | *cmlA, flo* | *cmlA, floR* | [45-47] |
|  | Antibiotic modification | acetyltransferases | *catA1, catB3* |  |

**References**

1. Brinas L, Zarazaga M, Saenz Y, Ruiz-Larrea F, Torres C. Beta-lactamases in ampicillin-resistant Escherichia coli isolates from foods, humans, and healthy animals. Antimicrob Agents Chemother. 2002;46(10):3156-63. PubMed PMID: 12234838; PubMed Central PMCID: PMCPMC128764.

2. Karami N, Hannoun C, Adlerberth I, Wold AE. Colonization dynamics of ampicillin-resistant Escherichia coli in the infantile colonic microbiota. J Antimicrob Chemother. 2008;62(4):703-8. doi: 10.1093/jac/dkn263. PubMed PMID: 18583327.

3. Wang J, Li Y, Xu X, Liang B, Wu F, Yang X, et al. Antimicrobial Resistance of Salmonella enterica Serovar Typhimurium in Shanghai, China. Front Microbiol. 2017;8:510. doi: 10.3389/fmicb.2017.00510. PubMed PMID: 28400764; PubMed Central PMCID: PMCPMC5368216.

4. Kariuki S, Okoro C, Kiiru J, Njoroge S, Omuse G, Langridge G, et al. Ceftriaxone-resistant Salmonella enterica serotype typhimurium sequence type 313 from Kenyan patients is associated with the blaCTX-M-15 gene on a novel IncHI2 plasmid. Antimicrob Agents Chemother. 2015;59(6):3133-9. doi: 10.1128/AAC.00078-15. PubMed PMID: 25779570; PubMed Central PMCID: PMCPMC4432211.

5. Canton R, Novais A, Valverde A, Machado E, Peixe L, Baquero F, et al. Prevalence and spread of extended-spectrum beta-lactamase-producing Enterobacteriaceae in Europe. Clin Microbiol Infect. 2008;14 Suppl 1:144-53. doi: 10.1111/j.1469-0691.2007.01850.x. PubMed PMID: 18154538.

6. Sheng WH, Badal RE, Hsueh PR, Program S. Distribution of extended-spectrum beta-lactamases, AmpC beta-lactamases, and carbapenemases among Enterobacteriaceae isolates causing intra-abdominal infections in the Asia-Pacific region: results of the study for Monitoring Antimicrobial Resistance Trends (SMART). Antimicrob Agents Chemother. 2013;57(7):2981-8. doi: 10.1128/AAC.00971-12. PubMed PMID: 23587958; PubMed Central PMCID: PMCPMC3697370.

7. Kiratisin P, Apisarnthanarak A, Laesripa C, Saifon P. Molecular characterization and epidemiology of extended-spectrum-beta-lactamase-producing Escherichia coli and Klebsiella pneumoniae isolates causing health care-associated infection in Thailand, where the CTX-M family is endemic. Antimicrob Agents Chemother. 2008;52(8):2818-24. doi: 10.1128/AAC.00171-08. PubMed PMID: 18505851; PubMed Central PMCID: PMCPMC2493136.

8. Liu X, Liu Y. Detection of plasmid-mediated AmpC beta-lactamase in Escherichia coli. Biomed Rep. 2016;4(6):687-90. doi: 10.3892/br.2016.661. PubMed PMID: 27284407; PubMed Central PMCID: PMCPMC4888007.

9. Reuland EA, Hays JP, de Jongh DM, Abdelrehim E, Willemsen I, Kluytmans JA, et al. Detection and occurrence of plasmid-mediated AmpC in highly resistant gram-negative rods. PLoS One. 2014;9(3):e91396. doi: 10.1371/journal.pone.0091396. PubMed PMID: 24642853; PubMed Central PMCID: PMCPMC3958353.

10. Jacoby GA. AmpC beta-lactamases. Clin Microbiol Rev. 2009;22(1):161-82, Table of Contents. doi: 10.1128/CMR.00036-08. PubMed PMID: 19136439; PubMed Central PMCID: PMCPMC2620637.

11. Suwantarat N, Carroll KC. Epidemiology and molecular characterization of multidrug-resistant Gram-negative bacteria in Southeast Asia. Antimicrob Resist Infect Control. 2016;5:15. doi: 10.1186/s13756-016-0115-6. PubMed PMID: 27148448; PubMed Central PMCID: PMCPMC4855802.

12. Matsumura Y, Peirano G, Motyl MR, Adams MD, Chen L, Kreiswirth B, et al. Global Molecular Epidemiology of IMP-Producing Enterobacteriaceae. Antimicrob Agents Chemother. 2017;61(4). doi: 10.1128/AAC.02729-16. PubMed PMID: 28167555; PubMed Central PMCID: PMCPMC5365671.

13. Nordmann P, Naas T, Poirel L. Global spread of Carbapenemase-producing Enterobacteriaceae. Emerg Infect Dis. 2011;17(10):1791-8. doi: 10.3201/eid1710.110655. PubMed PMID: 22000347; PubMed Central PMCID: PMCPMC3310682.

14. Girlich D, Poirel L, Szczepanowski R, Schluter A, Nordmann P. Carbapenem-hydrolyzing GES-5-encoding gene on different plasmid types recovered from a bacterial community in a sewage treatment plant. Appl Environ Microbiol. 2012;78(4):1292-5. doi: 10.1128/AEM.06841-11. PubMed PMID: 22156421; PubMed Central PMCID: PMCPMC3273036.

15. Queenan AM, Bush K. Carbapenemases: the versatile beta-lactamases. Clin Microbiol Rev. 2007;20(3):440-58, table of contents. doi: 10.1128/CMR.00001-07. PubMed PMID: 17630334; PubMed Central PMCID: PMCPMC1932750.

16. Evans BA, Amyes SG. OXA beta-lactamases. Clin Microbiol Rev. 2014;27(2):241-63. doi: 10.1128/CMR.00117-13. PubMed PMID: 24696435; PubMed Central PMCID: PMCPMC3993105.

17. Ge B, McDermott PF, White DG, Meng J. Role of efflux pumps and topoisomerase mutations in fluoroquinolone resistance in Campylobacter jejuni and Campylobacter coli. Antimicrob Agents Chemother. 2005;49(8):3347-54. doi: 10.1128/AAC.49.8.3347-3354.2005. PubMed PMID: 16048946; PubMed Central PMCID: PMCPMC1196287.

18. Hakanen A, Jalava J, Kotilainen P, Jousimies-Somer H, Siitonen A, Huovinen P. gyrA polymorphism in Campylobacter jejuni: detection of gyrA mutations in 162 C. jejuni isolates by single-strand conformation polymorphism and DNA sequencing. Antimicrob Agents Chemother. 2002;46(8):2644-7. PubMed PMID: 12121947; PubMed Central PMCID: PMCPMC127378.

19. Casas MR, Camargo CH, Soares FB, da Silveira WD, Fernandes SA. Presence of plasmid-mediated quinolone resistance determinants and mutations in gyrase and topoisomerase in Salmonella enterica isolates with resistance and reduced susceptibility to ciprofloxacin. Diagn Microbiol Infect Dis. 2016;85(1):85-9. doi: 10.1016/j.diagmicrobio.2016.01.016. PubMed PMID: 26971183.

20. Kim KY, Park JH, Kwak HS, Woo GJ. Characterization of the quinolone resistance mechanism in foodborne Salmonella isolates with high nalidixic acid resistance. Int J Food Microbiol. 2011;146(1):52-6. doi: 10.1016/j.ijfoodmicro.2011.01.037. PubMed PMID: 21354645.

21. Liu BT, Liao XP, Yang SS, Wang XM, Li LL, Sun J, et al. Detection of mutations in the gyrA and parC genes in Escherichia coli isolates carrying plasmid-mediated quinolone resistance genes from diseased food-producing animals. J Med Microbiol. 2012;61(Pt 11):1591-9. doi: 10.1099/jmm.0.043307-0. PubMed PMID: 22878251.

22. Korona-Glowniak I, Skrzypek K, Siwiec R, Wrobel A, Malm A. Fluoroquinolone-resistance mechanisms and phylogenetic background of clinical Escherichia coli strains isolated in south-east Poland. New Microbiol. 2016;39(3):210-5. PubMed PMID: 27455179.

23. Yugendran T, Harish BN. High incidence of plasmid-mediated quinolone resistance genes among ciprofloxacin-resistant clinical isolates of Enterobacteriaceae at a tertiary care hospital in Puducherry, India. PeerJ. 2016;4:e1995. doi: 10.7717/peerj.1995. PubMed PMID: 27168994; PubMed Central PMCID: PMCPMC4860338.

24. Nair S, Ashton P, Doumith M, Connell S, Painset A, Mwaigwisya S, et al. WGS for surveillance of antimicrobial resistance: a pilot study to detect the prevalence and mechanism of resistance to azithromycin in a UK population of non-typhoidal Salmonella. J Antimicrob Chemother. 2016;71(12):3400-8. doi: 10.1093/jac/dkw318. PubMed PMID: 27585964.

25. Phuc Nguyen MC, Woerther PL, Bouvet M, Andremont A, Leclercq R, Canu A. Escherichia coli as reservoir for macrolide resistance genes. Emerg Infect Dis. 2009;15(10):1648-50. doi: 10.3201/eid1510.090696. PubMed PMID: 19861064; PubMed Central PMCID: PMCPMC2866414.

26. Belanger AE, Shryock TR. Macrolide-resistant Campylobacter: the meat of the matter. J Antimicrob Chemother. 2007;60(4):715-23. doi: 10.1093/jac/dkm300. PubMed PMID: 17704515.

27. Gibreel A, Kos VN, Keelan M, Trieber CA, Levesque S, Michaud S, et al. Macrolide resistance in Campylobacter jejuni and Campylobacter coli: molecular mechanism and stability of the resistance phenotype. Antimicrob Agents Chemother. 2005;49(7):2753-9. doi: 10.1128/AAC.49.7.2753-2759.2005. PubMed PMID: 15980346; PubMed Central PMCID: PMCPMC1168676.

28. Qin S, Wang Y, Zhang Q, Zhang M, Deng F, Shen Z, et al. Report of ribosomal RNA methylase gene erm(B) in multidrug-resistant Campylobacter coli. J Antimicrob Chemother. 2014;69(4):964-8. doi: 10.1093/jac/dkt492. PubMed PMID: 24335515.

29. Ho PL, Leung LM, Chow KH, Lai EL, Lo WU, Ng TK. Prevalence of aminoglycoside modifying enzyme and 16S ribosomal RNA methylase genes among aminoglycoside-resistant Escherichia coli isolates. J Microbiol Immunol Infect. 2016;49(1):123-6. doi: 10.1016/j.jmii.2014.08.012. PubMed PMID: 25442860.

30. Ho PL, Wong RC, Lo SW, Chow KH, Wong SS, Que TL. Genetic identity of aminoglycoside-resistance genes in Escherichia coli isolates from human and animal sources. J Med Microbiol. 2010;59(Pt 6):702-7. doi: 10.1099/jmm.0.015032-0. PubMed PMID: 20185552.

31. Chen S, Zhao S, White DG, Schroeder CM, Lu R, Yang H, et al. Characterization of multiple-antimicrobial-resistant salmonella serovars isolated from retail meats. Appl Environ Microbiol. 2004;70(1):1-7. PubMed PMID: 14711619; PubMed Central PMCID: PMCPMC321239.

32. Zhang FY, Huo SY, Li YR, Xie R, Wu XJ, Chen LG, et al. A survey of the frequency of aminoglycoside antibiotic-resistant genotypes and phenotypes in Escherichia coli in broilers with septicaemia in Hebei, China. British poultry science. 2014;55(3):305-10. Epub 2014/02/28. doi: 10.1080/00071668.2014.891096. PubMed PMID: 24571237.

33. Cannatelli A, Giani T, Antonelli A, Principe L, Luzzaro F, Rossolini GM. First Detection of the mcr-1 Colistin Resistance Gene in Escherichia coli in Italy. Antimicrob Agents Chemother. 2016;60(5):3257-8. doi: 10.1128/AAC.00246-16. PubMed PMID: 26976865; PubMed Central PMCID: PMCPMC4862502.

34. Xavier BB, Lammens C, Ruhal R, Kumar-Singh S, Butaye P, Goossens H, et al. Identification of a novel plasmid-mediated colistin-resistance gene, mcr-2, in Escherichia coli, Belgium, June 2016. Euro Surveill. 2016;21(27). doi: 10.2807/1560-7917.ES.2016.21.27.30280. PubMed PMID: 27416987.

35. Quesada A, Ugarte-Ruiz M, Iglesias MR, Porrero MC, Martinez R, Florez-Cuadrado D, et al. Detection of plasmid mediated colistin resistance (MCR-1) in Escherichia coli and Salmonella enterica isolated from poultry and swine in Spain. Res Vet Sci. 2016;105:134-5. doi: 10.1016/j.rvsc.2016.02.003. PubMed PMID: 27033921.

36. Liu YY, Wang Y, Walsh TR, Yi LX, Zhang R, Spencer J, et al. Emergence of plasmid-mediated colistin resistance mechanism MCR-1 in animals and human beings in China: a microbiological and molecular biological study. Lancet Infect Dis. 2016;16(2):161-8. doi: 10.1016/S1473-3099(15)00424-7. PubMed PMID: 26603172.

37. Antunes P, Machado J, Sousa JC, Peixe L. Dissemination of sulfonamide resistance genes (sul1, sul2, and sul3) in Portuguese Salmonella enterica strains and relation with integrons. Antimicrob Agents Chemother. 2005;49(2):836-9. doi: 10.1128/AAC.49.2.836-839.2005. PubMed PMID: 15673783; PubMed Central PMCID: PMCPMC547296.

38. Shin HW, Lim J, Kim S, Kim J, Kwon GC, Koo SH. Characterization of trimethoprim-sulfamethoxazole resistance genes and their relatedness to class 1 integron and insertion sequence common region in gram-negative bacilli. J Microbiol Biotechnol. 2015;25(1):137-42. PubMed PMID: 25348695.

39. Blahna MT, Zalewski CA, Reuer J, Kahlmeter G, Foxman B, Marrs CF. The role of horizontal gene transfer in the spread of trimethoprim-sulfamethoxazole resistance among uropathogenic Escherichia coli in Europe and Canada. J Antimicrob Chemother. 2006;57(4):666-72. doi: 10.1093/jac/dkl020. PubMed PMID: 16464890.

40. El-Tayeb MA, Ibrahim ASS, Al-Salamah AA, Almaary KS, Elbadawi YB. Prevalence, serotyping and antimicrobials resistance mechanism of Salmonella enterica isolated from clinical and environmental samples in Saudi Arabia. Braz J Microbiol. 2017;48(3):499-508. doi: 10.1016/j.bjm.2016.09.021. PubMed PMID: 28245965; PubMed Central PMCID: PMCPMC5498448.

41. Labar AS, Millman JS, Ruebush E, Opintan JA, Bishar RA, Aboderin AO, et al. Regional dissemination of a trimethoprim-resistance gene cassette via a successful transposable element. PLoS One. 2012;7(5):e38142. doi: 10.1371/journal.pone.0038142. PubMed PMID: 22666464; PubMed Central PMCID: PMCPMC3364232.

42. Seputiene V, Povilonis J, Ruzauskas M, Pavilonis A, Suziedeliene E. Prevalence of trimethoprim resistance genes in Escherichia coli isolates of human and animal origin in Lithuania. J Med Microbiol. 2010;59(Pt 3):315-22. doi: 10.1099/jmm.0.015008-0. PubMed PMID: 20007760.

43. Karami N, Nowrouzian F, Adlerberth I, Wold AE. Tetracycline resistance in Escherichia coli and persistence in the infantile colonic microbiota. Antimicrob Agents Chemother. 2006;50(1):156-61. doi: 10.1128/AAC.50.1.156-161.2006. PubMed PMID: 16377681; PubMed Central PMCID: PMCPMC1346771.

44. Lu Y, Zhao H, Sun J, Liu Y, Zhou X, Beier RC, et al. Characterization of multidrug-resistant Salmonella enterica serovars Indiana and Enteritidis from chickens in Eastern China. PLoS One. 2014;9(5):e96050. doi: 10.1371/journal.pone.0096050. PubMed PMID: 24788434; PubMed Central PMCID: PMCPMC4008530.

45. Bischoff KM, White DG, Hume ME, Poole TL, Nisbet DJ. The chloramphenicol resistance gene cmlA is disseminated on transferable plasmids that confer multiple-drug resistance in swine Escherichia coli. FEMS microbiology letters. 2005;243(1):285-91. Epub 2005/01/26. doi: 10.1016/j.femsle.2004.12.017. PubMed PMID: 15668031.

46. Singer RS, Patterson SK, Meier AE, Gibson JK, Lee HL, Maddox CW. Relationship between phenotypic and genotypic florfenicol resistance in Escherichia coli. Antimicrob Agents Chemother. 2004;48(10):4047-9. Epub 2004/09/25. doi: 10.1128/AAC.48.10.4047-4049.2004. PubMed PMID: 15388477; PubMed Central PMCID: PMC521924.

47. Chuanchuen R, Padungtod P. Antimicrobial resistance genes in Salmonella enterica isolates from poultry and swine in Thailand. The Journal of veterinary medical science. 2009;71(10):1349-55. Epub 2009/11/06. PubMed PMID: 19887742.
