# Supplementary material for: Genotypic antimicrobial resistance assays for use on E. coli isolates and stool specimens
Source: PLoS One. 2019 May 10;14(5):e0216747. doi: 10.1371/journal.pone.0216747 (PMC6510447; doi:10.1371/journal.pone.0216747)
Supplement: S8 Table — (DOCX) [file pone.0216747.s008.docx]

**S8 Table. Subgroups or members of group assays.**

| **Assays** | **Detection subgroups/members** |
| --- | --- |
| CTX-M1 | CTX-M1,3,10-12,15,22-23,28-30,32-34,36-37,42,52-55,57-58,60-62,66,68-69,71-72,79-80,82,88,96,101,103,107,109,114,116,117,133,136,139,142 144,150,155-158,162-164,169-170,172 |
| CTX-M9 | CTX-M9,13-14,16-19,21,24,27,38,45-51,65,67,81,83-87,90,93,98-99,102,104-106,110-113,121-122,125-126,129-130,134,137,147-148,159,161 |
| CTX-M2-M74 | CTX-M2,4-7,20,31,43-44,56,59,76-77,92,95,97,115,124,131,141,165,171, CTX-M74,75 |
| CTX-M8-M25 | CTX-M8,40,63, CTX-M25-26,39,41,78,89,91,94,100,152,160 |
| PER | PER1-9 |
| VEB | VEB1-18 |
| CMY1-MOX | CMY1,8-11,19, MOX1-8 |
| FOX | FOX1-10 |
| CMY2-LAT | CMY2-7,12-18,20-27,36-48,49-51,53-87,90,93-95,98-108,110-119,131-133,135, LAT1-4 |
| ACT-MIR | ACT1-7,10,13-21,23-25,27,29-32,35-38, MIR1-17 |
| DHA | DHA1-3,5-7,9-10,12-22 |
| KPC | KPC1-19,22,24 |
| GES | GES1-27 |
| NDM | NDM1-13,15-16 |
| VIM | VIM1-6,8-19,23-29,30-39,42-46, |
| IMP | IMP1-15,17-21,23-26,28-30,32-34,37-38,40-45,47-49,51-56,59-63 |
| OXA-1 | OXA-1, 4, 30, 31 |
| OXA-48 | OXA162-163,181,199,204,232,244,245,247 |
| QnrA | QnrA1-8 |
| QnrS | QnrS1-9 |
| QnrB1 | QnrB1-3,5-7,9-10,13-20,23-24,26,29-30,32,36,40-52,54,56-59,61-62,64,66-68,70-72,74-82 |
| QnrB4 | QnrB4,8,22,55,65,39,11-12,21,25,27,28,33-35,37-38,60,69,73 |
| QepA | QepA1-4 |
| aac(3)-II | aac(3)-IIa-c-d-e |
| aph(3’)-I | aph(3’)-Ia-c |
| rmtB | rmtB1,2,3,4 |
| aadA1-2-17 | aadA1,2,3,8,12,13,15,17 |
| dfrA5-14 | dfrA5,14 |
| cmlA | cmlA1,4,5 |
