# Supplementary material for: Genotypic antimicrobial resistance assays for use on E. coli isolates and stool specimens
Source: PLoS One. 2019 May 10;14(5):e0216747. doi: 10.1371/journal.pone.0216747 (PMC6510447; doi:10.1371/journal.pone.0216747)
Supplement: S9 Table — (DOCX) [file pone.0216747.s009.docx]

**S9 Table.** **Analytical PCR performance of each assay on 384 well plate format.**

| **Assays** | **Linearity (R^2^)** | | **PCR efficiency (%)** | | **Assays** | **Linearity (R^2^)** | | **PCR efficiency (%)** | |
| --- | --- | --- | --- | --- | --- | --- | --- | --- | --- |
| Target1 / Target2 | Target1 | Target2 | Target1 | Target2 | Target1 / Target2 | Target1 | Target2 | Target1 | Target2 |
| TEM 104E / TEM 104K | 0.999 | 1.000 | 95 | 91 | gyrA87D-ESh^a^ / gyrA87NY-ESh^a^ | 0.998 | 1.000 | 95 | 93 |
| TEM 164R / TEM 164SC | 1.000 | 1.000 | 93 | 92 | parC80S-Sal^b^ / parC80I-Sal^b^ | 1.000 | 1.000 | 98 | 98 |
| PhHV / TEM 238S | 1.000 | 1.000 | 97 | 95 | parC80S-ESh^a^ / parC80I-ESh^a^ | 0.998 | 0.998 | 105 | 104 |
| SHV / SHV 238-240SE-SK | 1.000 | 0.999 | 94 | 101 | gyrA86T-Cj^c^ / gyrA86I-Cj^c^ | 0.999 | 0.998 | 95 | 94 |
| CTX-M1 / CTX-M8-M25 | 0.998 | 0.999 | 100 | 103 | gyrA86T-Cc^d^ / gyrA86I-Cc^d^ | 1.000 | 1.000 | 92 | 91 |
| CTX-M2-M74 / CTX-M9 | 0.998 | 0.999 | 98 | 101 | 23S-2075A -Cp^e^/ 23S-2075G-Cp^e^ | 1.000 | 1.000 | 99 | 99 |
| PER / VEB | 1.000 | 1.000 | 97 | 96 | ermB / mphA | 0.999 | 1.000 | 101 | 99 |
| CMY1-MOX / FOX | 1.000 | 0.999 | 95 | 102 | armA / rmtB | 1.000 | 1.000 | 95 | 93 |
| CMY2-LAT / ACT-MIR | 1.000 | 1.000 | 97 | 97 | aacC1 / aacC2 | 0.999 | 1.000 | 92 | 95 |
| DHA / none | 1.000 | NA | 94 | NA | aacC4 / aadB | 1.000 | 1.000 | 96 | 96 |
| KPC / GES | 1.000 | 1.000 | 95 | 94 | aphA1 / aadA1 | 0.999 | 0.999 | 96 | 96 |
| NDM / VIM | 0.999 | 0.997 | 98 | 104 | dfrA1 / dfrA12 | 1.000 | 1.000 | 97 | 97 |
| IMP / OXA-48 | 1.000 | 1.000 | 96 | 95 | dfrA5-14 / dfrA17 | 0.994 | 0.999 | 85 | 98 |
| OXA-1 / OXA-9 | 1.000 | 1.000 | 95 | 96 | sul1 / sul2 | 1.000 | 1.000 | 94 | 98 |
| QnrA / QnrS | 0.998 | 0.992 | 96 | 90 | sul3 / Bacterial 16S | 0.999 | 0.999 | 98 | 99 |
| QnrB1 / QnrB4 | 0.999 | 1.000 | 95 | 97 | tetA / tetB | 0.999 | 0.999 | 92 | 92 |
| aac(6’)-lb-104W/aac(6’)-lb-104R | 1.000 | 1.000 | 96 | 94 | catA1 / catB3 | 0.996 | 1.000 | 109 | 99 |
| gyrA87G-ESh^a^ / aac(6’)-lb-181Y | 1.000 | 1.000 | 95 | 96 | cmlA / floR | 0.991 | 0.985 | 91 | 85 |
| QepA / gyrA87G-Sal^b^ | 0.999 | 0.998 | 98 | 98 | mcr-1 / mcr-2 | 0.999 | 0.999 | 96 | 96 |
| gyrA83S-Sal^b^ / gyrA83FY-Sal^b^ | 0.999 | 1.000 | 92 | 92 | *E.coli-Shigella* spp./*Shigella* spp. | 0.999 | 1.000 | 100 | 96 |
| gyrA87D-Sal^b^ / gyrA87NY-Sal^b^ | 1.000 | 1.000 | 98 | 99 | *Salmonella* spp. / *C. jejuni-coli* | 1.000 | 0.999 | 99 | 99 |
| gyrA83S-ESh^a^ / gyrA83L-ESh^a^ | 1.000 | 1.000 | 91 | 90 |  |  |  |  |  |
| Average ± SD | 0.999 ± 0.002 (96.2 ± 3.9) | | | | | | | | |

NA; not applicable, this assay is singleplex

^a^ESh ; *E.coli-Shigella* spp., ^b^ Sal ; *Salmonella* spp., ^c^ Cj ; *C. jejuni*, ^d^ Cc ; *C. coli*, ^e^Cp ; *Campylobacter* spp.
