# Supplementary material for: Genotypic antimicrobial resistance assays for use on E. coli isolates and stool specimens
Source: PLoS One. 2019 May 10;14(5):e0216747. doi: 10.1371/journal.pone.0216747 (PMC6510447; doi:10.1371/journal.pone.0216747)
Supplement: S10 Table — (DOCX) [file pone.0216747.s010.docx]

**S10 Table.** **Analytical performance of antimicrobial resistance TaqMan array card (AMR-TAC).**

| **Assays** | **Linearity (R^2^)** | | **PCR efficiency (%)** | | **LOD^a^**  **(copies no./rxn)** | | **Low- and high-conc. CV (%) for^b^ :** | | | |
| --- | --- | --- | --- | --- | --- | --- | --- | --- | --- | --- |
|  |  |  |  |  |  |  | **Repeatability** | | **Reproducibility** | |
| **Target1 / Target2** | **Target1** | **Target2** | **Target1** | **Target2** | **Target1** | **Target2** | **Target1** | **Target2** | **Target1** | **Target2** |
| TEM 104E / TEM 104K | 0.999 | 0.997 | 93 | 92 | 10^4^ (10) | 10^4^ (10) | 1.1, 2.1 | 3.0, 3.8 | 4.0, 5.1 | 3.0, 6.0 |
| TEM 164R / TEM 164SC | 1.000 | 1.000 | 95 | 93 | 10^4^ (10) | 10^4^ (10) | 1.6, 1.8 | 2.4, 2.3 | 1.6, 2.5 | 2.3, 3.2 |
| PhHV / TEM 238S | 1.000 | 0.999 | 93 | 94 | 10^4^ (10) | 10^4^ (10) | 3.1, 4.2 | 1.4, 4.8 | 3.7, 4.1 | 6.3, 4.8 |
| SHV / SHV 238-240SE-SK | 1.000 | 0.999 | 96 | 94 | 10^4^ (10) | 10^4^ (10) | 1.9, 0.7 | 2.3, 0.9 | 3.6, 8.5 | 2.5, 3.3 |
| CTX-M1 / CTX-M8-M25 | 1.000 | 1.000 | 95 | 92 | 10^4^ (10) | 10^4^ (10) | 3.1, 3.9 | 2.0, 2.3 | 3.8, 8.0 | 3.2, 5.1 |
| CTX-M2-M74 / CTX-M9 | 1.000 | 1.000 | 98 | 100 | 10^4^ (10) | 10^4^ (10) | 1.5, 1.1 | 2.6, 3.6 | 2.8, 4.3 | 7.3, 7.6 |
| PER / VEB | 1.000 | 1.000 | 96 | 98 | 10^4^ (10) | 10^4^ (10) | 2.2, 6.9 | 3.2, 8.9 | 3.3, 5.7 | 6.5, 6.9 |
| CMY1-MOX / FOX | 1.000 | 1.000 | 89 | 93 | 10^4^ (10) | 10^4^ (10) | 8.8, 1.8 | 1.3, 2.9 | 8.3, 2.7 | 2.4, 3.6 |
| CMY2-LAT / ACT-MIR | 1.000 | 1.000 | 94 | 95 | 10^4^ (10) | 10^4^ (10) | 4.1, 3.9 | 4.2, 3.3 | 5.1, 8.2 | 4.1, 7.6 |
| DHA / none | 0.999 | ND | 94 | ND | 10^4^ (10) | ND | 1.3, 4.9 | ND | 2.8, 6.2 | ND |
| KPC / GES | 1.000 | 1.000 | 96 | 95 | 10^4^ (10) | 10^4^ (10) | 2.8, 7.7 | 1.3, 6.7 | 4.2, 6.2 | 3.6, 6.3 |
| NDM / VIM | 0.999 | 0.999 | 96 | 99 | 10^4^ (10) | 10^4^ (10) | 3.4, 8.5 | 3.3, 6.2 | 4.2, 6.0 | 6.5, 7.3 |
| IMP / OXA-48 | 1.000 | 1.000 | 100 | 97 | 10^4^ (10) | 10^4^ (10) | 7.4, 9.5 | 5.0, 6.7 | 4.3, 10.7 | 3.3, 6.3 |
| OXA-1 / OXA-9 | 1.000 | 1.000 | 97 | 95 | 10^4^ (10) | 10^4^ (10) | 2.2, 1.9 | 1.4, 2.2 | 3.1, 6.0 | 2.1, 5.1 |
| QnrA / QnrS | 1.000 | 1.000 | 95 | 95 | 10^4^ (10) | 10^4^ (10) | 2.8, 3.8 | 3.3, 6.9 | 2.6, 3.3 | 2.3, 3.9 |
| QnrB1 / QnrB4 | 1.000 | 1.000 | 93 | 93 | NA | NA | 4.0, 5.0 | 4.0, 5.6 | 5.1, 6.7 | 6.7, 3.8 |
| aac(6’)-lb-104W / 104R | 0.999 | 0.999 | 94 | 94 | 10^4^ (10) | 10^4^ (10) | 3.4, 6.8 | 4.7, 7.1 | 2.9, 5.3 | 3.9, 5.3 |
| gyrA87G-ESh^c^ / aac(6’)-lb-181Y | 1.000 | 1.000 | 96 | 95 | 10^4^ (10) | 10^4^ (10) | 3.2, 6.3 | 3.8, 1.7 | 4.5, 3.9 | 2.0, 1.8 |
| QepA / gyrA87G-Sal^d^ | 1.000 | 1.000 | 96 | 96 | 10^4^ (10) | 10^4^ (10) | 2.0, 2.0 | 2.4, 3.0 | 3.0, 2.9 | 2.3, 3.2 |
| gyrA83S-Sal^d^ / gyrA83FY-Sal^d^ | 0.999 | 1.000 | 94 | 94 | 10^4^ (10) | 10^4^ (10) | 3.1, 1.4 | 2.9, 1.2 | 1.7, 2.2 | 2.8, 2.2 |
| gyrA87D-Sal^d^ / gyrA87NY-Sal^d^ | 1.000 | 0.999 | 94 | 97 | 10^4^ (10) | 10^4^ (10) | 3.3, 2.8 | 4.7, 4.0 | 3.6, 6.4 | 6.1, 11.3 |
| gyrA83S-ESh^c^ / gyrA83L-ESh^c^ | 0.999 | 1.000 | 93 | 95 | NA | 10^4^ (10) | 1.8, 4.9 | 2.2, 2.5 | 3.4, 4.7 | 1.9, 2.4 |
| gyrA87D-ESh^c^ / 87NY-ESh^c^ | 0.996 | 0.997 | 88 | 90 | NA | 10^4^ (10) | 2.9, 7.1 | 6.9, 6.1 | 3.1, 4.3 | 5.6, 8.8 |
| parC80S-Sal^d^ / parC80I-Sal^d^ | 0.999 | 0.999 | 92 | 91 | 10^4^ (10) | 10^4^ (10) | 1.0, 1.4 | 1.5, 1.5 | 2.1, 2.5 | 2.5, 2.6 |
| parC80S-ESh^c^ / parC80I-ESh^c^ | 0.999 | 0.998 | 94 | 97 | NA | 10^4^ (10) | 0.5, 1.2 | 2.2, 1.6 | 2.1, 2.6 | 2.3, 3.2 |
| gyrA86T-Cj^e^ / gyrA86I-Cj^e^ | 1.000 | 0.999 | 94 | 96 | 10^4^ (10) | 10^4^ (10) | 1.7, 2.4 | 4.2, 1.2 | 2.2, 4.8 | 1.9, 5.4 |
| gyrA86T-Cc^f^ / gyrA86I-Cc^f^ | 1.000 | 0.999 | 95 | 93 | 10^4^ (10) | 10^4^ (10) | 2.8, 1.5 | 2.3, 1.1 | 2.5, 3.3 | 4.7, 2.4 |
| 23S-2075A -Cp^g^ / 23S-2075G-Cp^g^ | 0.999 | 0.999 | 95 | 93 | NA | NA | 1.5, 4.8 | 1.6, 1.8 | 4.9, 5.2 | 1.9, 2.9 |
| ermB / mphA | 1.000 | 1.000 | 96 | 98 | NA | 10^4^ (10) | 2.4, 2.3 | 3.7, 1.6 | 5.9, 5.9 | 7.2, 5.7 |
| armA / rmtB | 0.999 | 1.000 | 97 | 96 | 10^4^ (10) | 10^4^ (10) | 7.1, 7.1 | 3.2, 0.8 | 4.9, 6.2 | 2.6, 1.9 |
| aacC1 / aacC2 | 1.000 | 0.999 | 95 | 97 | 10^4^ (10) | 10^4^ (10) | 2.8, 2.5 | 2.8, 4.2 | 3.3, 4.7 | 3.5, 5.3 |
| aacC4 / aadB | 0.999 | 1.000 | 99 | 100 | 10^4^ (10) | 10^4^ (10) | 1.3, 8.5 | 1.9, 4.6 | 5.5, 5.2 | 6.6, 4.5 |
| aphA1 / aadA1-2-17 | 1.000 | 1.000 | 95 | 93 | NA | NA | 2.1, 3.3 | 2.9, 3.2 | 4.1, 5.9 | 3.5, 4.3 |
| dfrA1 / dfrA12 | 0.999 | 1.000 | 96 | 95 | 10^4^ (10) | 10^4^ (10) | 2.3, 6.1 | 1.7, 6.7 | 5.5, 5.2 | 6.5, 4.3 |
| dfrA5-14 / dfrA17 | 1.000 | 1.000 | 94 | 96 | 10^4^ (10) | 10^4^ (10) | 3.0, 5.3 | 3.4, 6.1 | 3.9, 3.6 | 5.7, 5.8 |
| sul1 / sul2 | 0.999 | 0.999 | 94 | 96 | NA | NA | 5.6, 6.2 | 3.9, 9.0 | 5.5, 7.8 | 5.5, 8.3 |
| sul3 / Bacterial 16S | 1.000 | 1.000 | 97 | 98 | NA | NA | 5.5, 5.4 | 5.5, 6.5 | 5.4, 7.3 | 3.2, 6.1 |
| tetA / tetB | 1.000 | 1.000 | 94 | 97 | NA | NA | 1.1, 2.1 | 1.9, 4.5 | 1.6, 3.0 | 1.1, 4.1 |
| catA1 / catB3 | 1.000 | 1.000 | 95 | 97 | NA | 10^4^ (10) | 2.7, 3.4 | 3.4, 2.8 | 5.1, 6.6 | 5.3, 5.6 |
| cmlA / floR | 0.999 | 0.999 | 102 | 99 | NA | NA | 1.5, 3.6 | 1.5, 5.7 | 2.5, 3.2 | 3.6, 3.8 |
| mcr-1 / mcr-2 | 1.000 | 1.000 | 94 | 95 | 10^4^ (10) | 10^4^ (10) | 2.8, 6.1 | 2.6, 6.6 | 4.0, 3.2 | 5.2, 5.2 |
| *E.coli-Shigella* spp./*Shigella* spp. | 1.000 | 1.000 | 92 | 93 | NA | 10^4^ (10) | 5.3, 7.6 | 4.7, 8.2 | 12.3, 9.4 | 10.4, 10.2 |
| *Salmonella* spp. / *C. jejuni-coli* | 1.000 | 1.000 | 93 | 95 | 10^4^ (10) | 10^4^ (10) | 4.7, 5.3 | 5.3, 4.9 | 3.4, 5.9 | 5.2, 4.1 |
| Average ± SD | 0.999 ± 0.001 | | 95.1 ± 2.5 | |  |  | 3.6 ± 2.0 | | 4.7 ± 2.1 | |

^a^LOD is lowest copies number of positive control plasmid spiked into 200 mg of stool that were 100% detectable with 10 distinct extractions/amplification

^b^Coefficients of variance (CV) of Ct value at both low (LOD) and high (100-fold higher than LOD) spiked concentrations are shown

^c^ESh ; *E.coli-Shigella* spp., ^d^ Sal ; *Salmonella* spp., ^e^ Cj ; *C. jejuni*, ^f^ Cc ; *C. coli*, ^g^Cp ; *Campylobacter* spp.

ND; not done, this assay is singleplex

NA; not applicable, according to the donor stool harbor those gene as an background result to unable to determine LOD
