# Supplementary figures and images for: Genotypic antimicrobial resistance assays for use on E. coli isolates and stool specimens
Source: PLoS One. 2019 May 10;14(5):e0216747. doi: 10.1371/journal.pone.0216747 (PMC6510447; doi:10.1371/journal.pone.0216747)

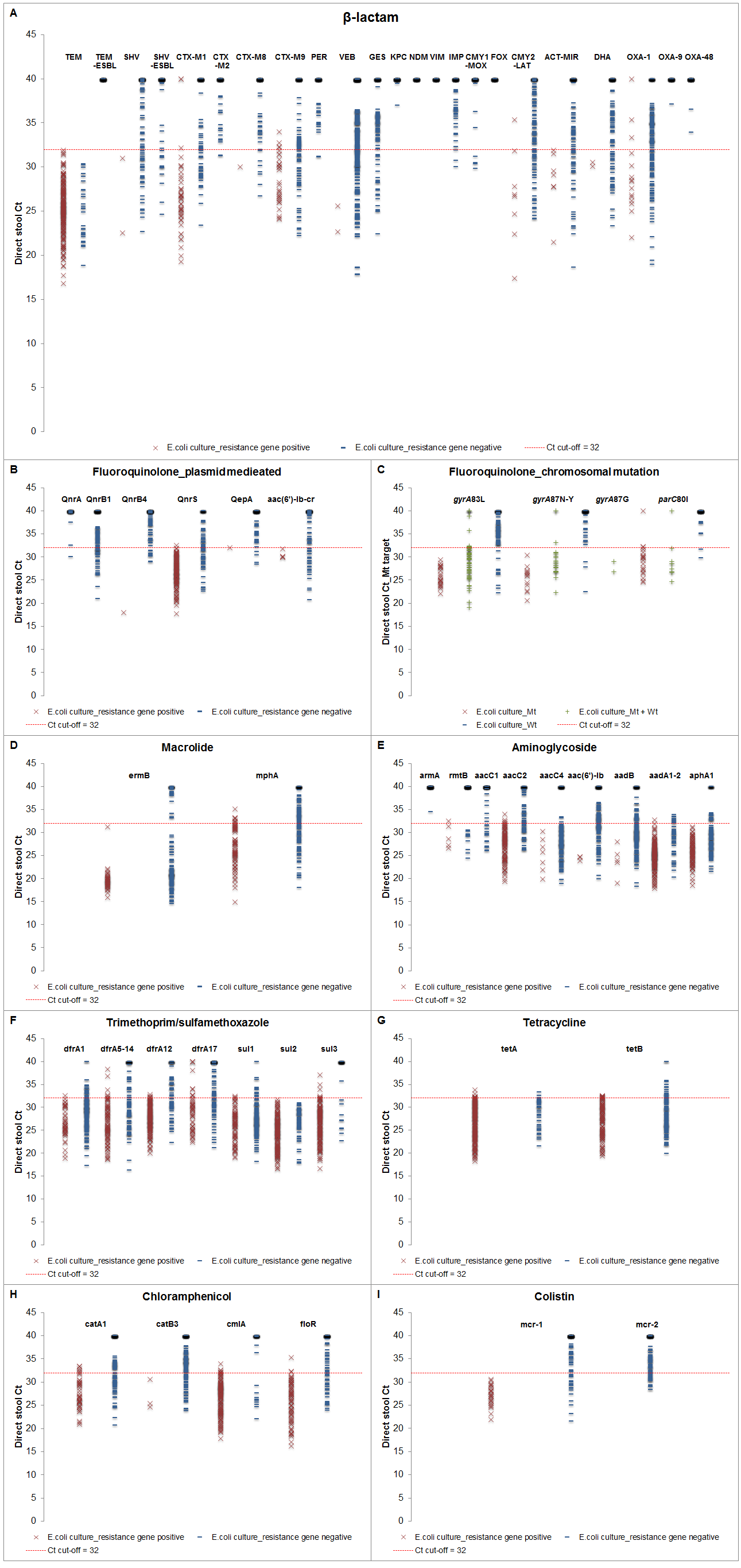

Supplement: S1 Fig — Scatter plot of difference Ct values of 220 direct stools against paired E. coli isolates results of each target gene associated resistance to β-lactam (A), fluoroquinolone (B and C), Macrolide (D), aminoglycoside (E), trimethoprim/sulfamethoxazole (F), tetracycline (G), chloramphenicol (H), and colistin (I). Receiver Operating Curves (ROC) identified cut-off for optimized positive/negative categorization of direct stool against E. coli culture isolates for E. coli specific gene gyrA and parC, then the same cut-off was applied to all other gene targets which non-E. coli specific. (TIF) [file pone.0216747.s011.tif]
